# Repurposing FDA‐Approved Drugs to Inhibit Fungal PPTases for Broad‐Spectrum Synergy

**DOI:** 10.1002/advs.77354

**Published:** 2026-08-21

**Authors:** Zili Song, Can Zhao, Qiaoling Wang, Hongjiao Zhang, Xiao Liu, Linqi Wang, Michael Bromley, Wen‐Bing Yin

**Affiliations:** ^1^ State Key Laboratory of Microbial Diversity and Innovative Utilization Institute of Microbiology Chinese Academy of Sciences Beijing China; ^2^ Medical School University of Chinese Academy of Sciences Beijing China; ^3^ School of Biological and Chemical Sciences Manchester Metropolitan University Manchester UK; ^4^ Manchester Fungal Infection Group, Division of Infection, Immunity and Respiratory Medicine University of Manchester Manchester UK; ^5^ School of Life Sciences and Medicine University of Science and Technology of China Hefei China

**Keywords:** antifungal drug, combination therapy, pan‐fungal target, pathogenic fungi, PPTase, secondary metabolism

## Abstract

Fungal secondary metabolites serve as crucial virulence effectors; however, the potential of their biosynthetic pathways as antifungal targets remains inadequately comprehended. In this study, we put forward a novel antifungal strategy that targets the conserved global secondary metabolic pathway in pathogenic fungi. Through genome mining, phylogenetic analysis, and structural modeling, we identified invariant residues in phosphopantetheinyl transferases (PPTases), which are essential for secondary metabolism, and revealed them as pan‐fungal targets. High‐throughput screening indicated that the FDA‐approved tepotinib and eltrombopag bind to PPTases to interfere with mycotoxin biosynthesis, thereby reducing the fungal burden and alleviating lung inflammation in a mouse model of pulmonary aspergillosis. These compounds exhibited broad‐spectrum activity against common pathogens, including *Aspergillus fumigatus*, *Mucor circinelloides*, *Aspergillus flavus*, *Fusarium oxysporum*, *Cryptococcus neoformans*, and *Cryptococcus gattii*. Notably, both drug candidates demonstrated synergistic effects with amphotericin B, reducing its half‐maximal inhibitory concentration by 7.4‐ and 2.6‐fold, respectively. This research provides a metabolic targeting framework and paves new paths for the rational design of broad‐spectrum antifungals and combination therapies.

## Graphical abstract

Fungal secondary metabolites serve as crucial virulence effectors; however, the potential of their biosynthetic pathways as antifungal targets remains inadequately comprehended. Repurposed FDA‐approved drugs inhibit fungal PPTase (essential for secondary metabolism) in vivo. This study establishes a metabolism‐targeted “disarm rather than kill” antifungal strategy, offering promising leads for new broad‐spectrum antifungals and combinatorial treatments.

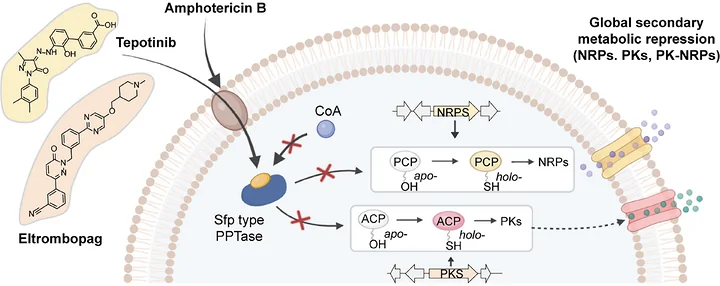

## 1 Introduction

Fungal infections have emerged as an increasingly severe global health burden, resulting in approximately 3.75 million annual deaths [1, 2]. This crisis is further exacerbated by the growing population of immunocompromised individuals and the excessive use of antifungals in agricultural, veterinary, and clinical contexts [3, 4, 5]. Currently, only three drug classes (azoles, polyenes, echinocandins) remain clinically available [6, 7]. However, their clinical utility is restricted by several insurmountable obstacles, including human toxicity (e.g., nephrotoxicity of amphotericin B), narrow antimicrobial spectra, pharmacokinetic conflicts with chemotherapeutics, and rapidly evolving resistance, rendering some infections untreatable [8, 9]. A more profound challenge lies in the fact that all three classes target conserved fungal cell membrane/wall pathways [6, 10]. As eukaryotes, fungi share highly conserved molecular components with humans [10, 11], and this evolutionary commonality makes the identification of fungal‐specific pathways with both specificity and druggability a core dilemma in the field.

Traditional antifungal drug discovery predominantly relies on growth‐inhibition screens, while largely overlooking fungal metabolic processes. In contrast, fungal secondary metabolites (SMs) present an untapped opportunity. These compounds serve as crucial defensive “weapons” that enable fungi to adapt to environmental stress and interact with the host immune system [12, 13]. They also significantly contribute to fungal virulence [14, 15] and exhibit structural characteristics distinct from mammalian metabolites. Among these SMs, mycotoxins attract particular attention due to their toxicity, carcinogenicity, and/or immunosuppressive activity in mammals. Representative examples include DHN‐melanin, which confers the ability to resist reactive oxygen species (ROS) [16, 17], gliotoxin that impairs immune cell function [18, 19], fumiquinazoline C that inhibits macrophage phagocytosis [20], and siderophores that compete with the host for trace elements [21, 22]. Notably, the biosynthetic pathways of fungal SMs follow a conserved logic, highlighting it as a potential pan‐antifungal target.

Fungal SM biosynthesis proceeds via a modular “assembly line” mechanism that primarily generates polyketides (PKs) and non‐ribosomal peptides (NRPs) [23, 24]. Each module possesses a conserved thiolation domain, and 4’‐phosphopantetheinyl transferases (PPTases) initiate metabolic activation by transferring phosphopantetheine from coenzyme A to acyl carrier proteins. This modification transforms inactive *apo*‐forms into functional *holo*‐forms, which are essential for PK and NRP biosynthesis [25]. In *Aspergillus fumigatus*, the sfp‐type PPTase PptA is non‐redundant for most PKs and NRPs biosynthesis [26, 27]. Consistently, the *pptA* deletion mutant exhibits elevated requirements for lysine and iron that exceed the nutrient availability in mammalian tissues, ultimately leading to a complete attenuation of virulence in both insect and murine infection models [28]. Despite these findings, the potential of PptA as a therapeutic target for SM‐related antifungal strategies remains largely unexplored, particularly regarding its structural conservation among pathogenic fungi and the feasibility of developing targeted small‐molecule inhibitors.

Here, we propose an alternative anti‐infective strategy that targets the global secondary metabolism of pathogenic fungi. Through the integration of genome mining, phylogenetic analysis, and structural modeling, we identified conserved binding‐pocket residues across pathogenic fungal PPTases, suggesting PPTases as a promising pan‐fungal therapeutic target. High‐throughput screening of the FDA‐approved drug library, combined with computational simulations along with subsequent in vitro and in vivo activity evaluations, revealed that the antitumor agent tepotinib and the non‐peptide thrombopoietin receptor agonist eltrombopag possess potent antifungal properties. Both compounds significantly reduce fungal colonization and alleviate murine lung tissue damage. Mechanistically, isothermal titration calorimetry (ITC) assays with purified PptA demonstrated that tepotinib and eltrombopag bind to the active sites on PptA, directly interfering with mycotoxin biosynthesis. Moreover, these two compounds exhibit broad‐spectrum antifungal activity against a range of clinical *A. fumigatus* isolates, other filamentous fungi including *Mucor circinelloides*, *Aspergillus flavus*, and *Fusarium oxysporum*, as well as pathogenic *Cryptococcus* species such as *Cryptococcus neoformans* and *Cryptococcus gattii*. Critically, they synergize with amphotericin B, reducing its IC_50_ by 7.4‐ and 2.6‐fold, respectively. This work not only provides drug candidates for combination therapies but also establishes a metabolic targeting framework, opening new avenues for combating fungal infections.

## 2 Results

### 2.1 Discovery of Candidate Antifungal Drugs Targeting Fungal Secondary Metabolic Biosynthetic Pathways

Fungal secondary metabolites (SMs) function as crucial virulence effectors. Non‐ribosomal peptides (NRPs) and polyketides (PKs) have emerged as the core classes driving pathogenicity [14], accounting for 74% of all 462,654 biosynthetic gene clusters (BGCs) in the fungal kingdom (Figure S1). Notably, in *A. fumigatus* A1163, *A. flavus* NRRL3357, *M. circinelloides* 1006PhL, and *F. oxysporum* Fo47, these two types of BGCs account for 77%, 73%, 63%, and 67%, respectively (Table S1). This discovery underscores that the shared biosynthetic pathways of these two major classes are promising targets for antifungal intervention. Functional annotation of the shared NRP/PK biosynthetic pathways highlights the sfp‐type 4’‐phosphopantetheinyl transferase (PPTase). This enzyme catalyzes the transfer of a phosphopantetheine moiety from coenzyme A (CoA) to the serine residues of peptidyl carrier proteins (PCPs) or acyl carrier proteins (ACPs), thereby activating these scaffolds for “assembly‐line” biosynthesis (Figure 1A, Figure S2).

**FIGURE 1:**
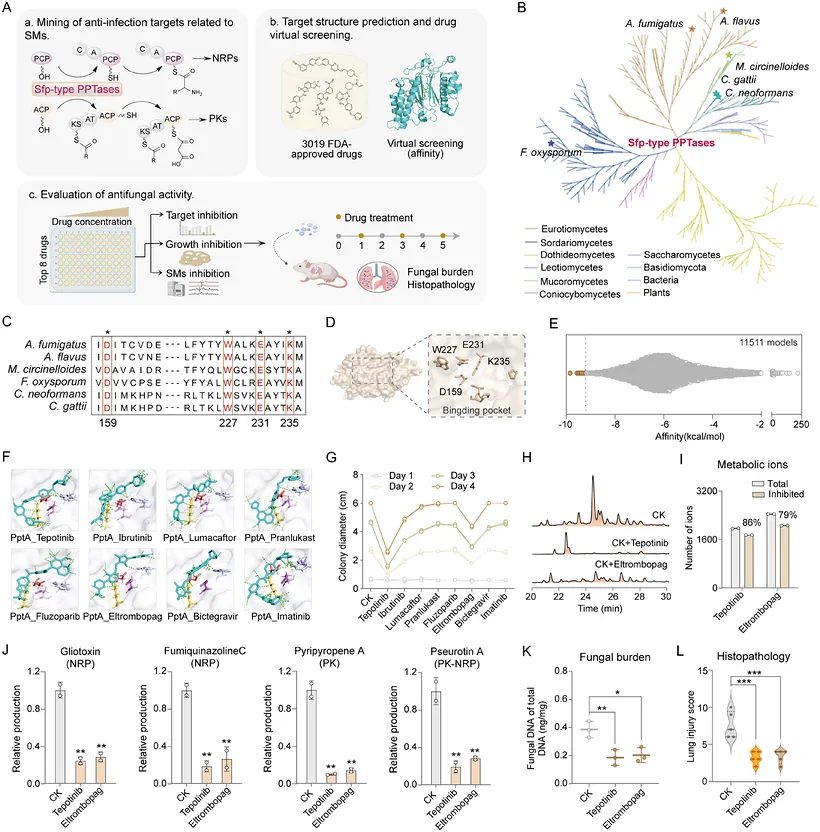
Mining FDA‐approved drugs by targeting Sfp‐type PPTases for antifungal therapy. (A) Workflow of drug screening and evaluation against *A. fumigatus*. SMs: Secondary metabolites. Sfp‐type PPTases: 4’‐phosphopantetheinyl transferases. (B) Phylogenetic analysis of the sfp‐type PPTases in fungi. (C) Conservative analysis of the binding sites in the pathogenic fungal sfp‐type PPTases. Binding sites D159, W227, E231, and K235 of the two drug candidates were conserved in fungal sfp‐type PPTases. (D) Structure and binding pocket prediction of sfp‐type PPTase PptA in *A. fumigatus*. (E) Virtual screening of FDA‐approved drugs targeting *A. fumigatus* PptA via molecular docking. A total of 11 511 structural models were obtained by docking PptA with 3,019 FDA‐approved drugs. Brown dots indicate the 8 models with strong binding affinity. (F) Interaction models of the top 8 FDA‐approved drugs with high affinity to PptA. Gray: W227. Red: D159. Purple: E231. Yellow: K235. Cyan: Candidate drugs. (G) Activity evaluation of the top 8 drugs with high affinity to PptA against *A. fumigatus*. (H) Metabolic profiles of *A. fumigatus* at 254 nm after treatment with tepotinib and eltrombopag. (I, J) Metabolomic analysis of total metabolic ion products and typical secondary metabolites inhibited by tepotinib and eltrombopag. The biosynthesis of mycotoxins, including nonribosomal peptides (gliotoxin and fumiquinazoline C), polyketides (pyripyropene A), and hybrid metabolites (pseurotin A), was significantly inhibited by the drugs. A Log_2_foldchange < −1 indicates repressed metabolic ions in drug‑treated groups compared with the control. (K) Pulmonary fungal burden in a mouse model of invasive aspergillosis after 5 days of treatment with two drug candidates (*n* = 3). (L) Histopathological assessment in the mouse lungs after 5 days of treatment with two drug candidates (*n* = 5). Quantification of lung tissue injury was performed according to the Smith scoring criteria. All error bars are expressed as mean ± SD. Statistical analysis was performed using Two‐way ANOVA (Significant at ^*^ *p* < 0.05, ^**^ *p* < 0.01, ^***^ *p* < 0.001).

Given the widespread presence of SMs across fungal lineages and the conservation of their biosynthetic machinery, a comprehensive phylogenetic analysis of sfp‐type PPTase orthologs was conducted (Table S1). The results indicated that sfp‐type PPTase homologs are widely distributed throughout the fungal kingdom, with significant representation in highly pathogenic species such as *A. fumigatus*, *A. flavus*, *M. circinelloides*, *F. oxysporum*, and the basidiomycetes *C. gattii* and *C. neoformans* (Figure 1B, Figure S3). Further sequence alignment identified four evolutionarily invariant amino acid residues (D159, W227, E231, and K235), which present a potential target for the development of broad‐spectrum antifungal agents (Figure 1C, Figure S4). In *A. fumigatus*, this enzyme is named PptA [26, 28, 29]. To exploit this conserved target, the three‐dimensional structure of PptA was predicted using AlphaFold3 (Figure S5), followed by virtual screening of 3,019 marketed drugs from the FDA‐Approved Drug Library (Figure 1D,E). Drug candidates were prioritized based on their binding affinity to the conserved active site of PptA, generating 11,511 unique binding conformations (Table S2). The top 8 hits—tepotinib, ibrutinib, lumacaftor, pranlukast, fluzoparib, eltrombopag, bictegravir, and imatinib—exhibited strong binding affinities exceeding ‐9 kcal/mol (Figure 1F, Figures S6 and S7).

Subsequently, the in vitro antifungal efficacy of these candidates against *A. fumigatus* was evaluated at 200 µM. Tepotinib displayed significant growth inhibition with an inhibition rate of 67%, while eltrombopag showed moderate inhibitory activity (36%) (Figure 1G). Further analysis of the PptA–tepotinib and PptA–eltrombopag binding models revealed distinct intermolecular interaction patterns. Tepotinib engages D159, K235 and W227 via hydrogen bonds, cation‑*π* contacts and *π*‑‐*σ* interactions, respectively. By contrast, eltrombopag forms hydrogen bonds, *π*‑‐*π* stacking and cation‑*π* interactions with W227, E231 and K235 correspondingly (Figure S8). Strikingly, the production of fungal metabolites was significantly suppressed after treatment with the two candidate drugs at 200 µM (Figure 1H, Figure S9). Further metabolomics analysis of LC‐MS‐acquired ion products was performed using a threshold of Log_2_foldchange < −1 to define inhibited metabolites (Figure S10). In total, 2,030 ions were detected in the tepotinib‐treated group, with 1,747 (86%) inhibited, while 2,602 ions were identified in the eltrombopag‐treated group, with 2062 (79%) inhibited (Figure 1I, Table S3). Specifically, the levels of representative virulence‐associated metabolites, including gliotoxin (NRP), fumiquinazoline C (NRP), pyripyropene A (PK), and pseurotin A (PK‐NRP hybrid), were markedly suppressed. Their abundances decreased by 76%, 81%, 90%, and 81% upon tepotinib treatment, and by 71%, 74%, 86%, and 72% following eltrombopag treatment, respectively (Figure 1J). These two candidate drugs inhibit metabolism far more potently than growth, highlighting their preferential suppression of fungal metabolic processes.

In a murine model of invasive aspergillosis, 5‐day intranasal intermittent administration of tepotinib and eltrombopag reduced pulmonary fungal burden by 2.1‐ and 1.9‐fold, respectively, and significantly alleviated lung tissue damage (Figure 1K,L, Figure S11). Collectively, these findings suggest that targeting PPTase within the fungal secondary metabolic biosynthetic pathway, which modulates both fungal virulence and growth, represents a potential therapeutic strategy against fungal infections.

### 2.2 Identification of Candidate Antifungal Drug Targets in *A. fumigatus*

To identify the potential targets of tepotinib and eltrombopag, a *pptA* deletion mutant was generated in the *A. fumigatus* A1163 background (Figure S12). Phenotypic assessment of the Δ*pptA* mutant showed substantial impairment of hyphal elongation and asexual sporulation (Figure 2A, Figure S12D). Subsequent HPLC results indicated that metabolic activity was largely suppressed in the Δ*pptA* mutant (Figure 2B). Total ion peaks offer a reliable indication of the relative quantity of metabolites produced (Table S4). Principal components analysis (PCA) also revealed significant metabolic disparities between the wild‐type and the Δ*pptA* mutant (Figure S12E). Further comparative metabolomics analysis suggested that the deletion of *pptA* reduced the biosynthesis of the majority of metabolites, with approximately 90% being inhibited (Figure 2C,D, Figure S12F). Notably, key virulence‐related SMs, including gliotoxin and pyripyropene A, were scarcely detectable in the Δ*pptA* mutant (Figure 2E). The absence of these key virulence‐associated metabolites is highly consistent with the metabolic inhibition observed during drug treatment, implying a potential association between the candidate drugs and the PptA protein.

**FIGURE 2:**
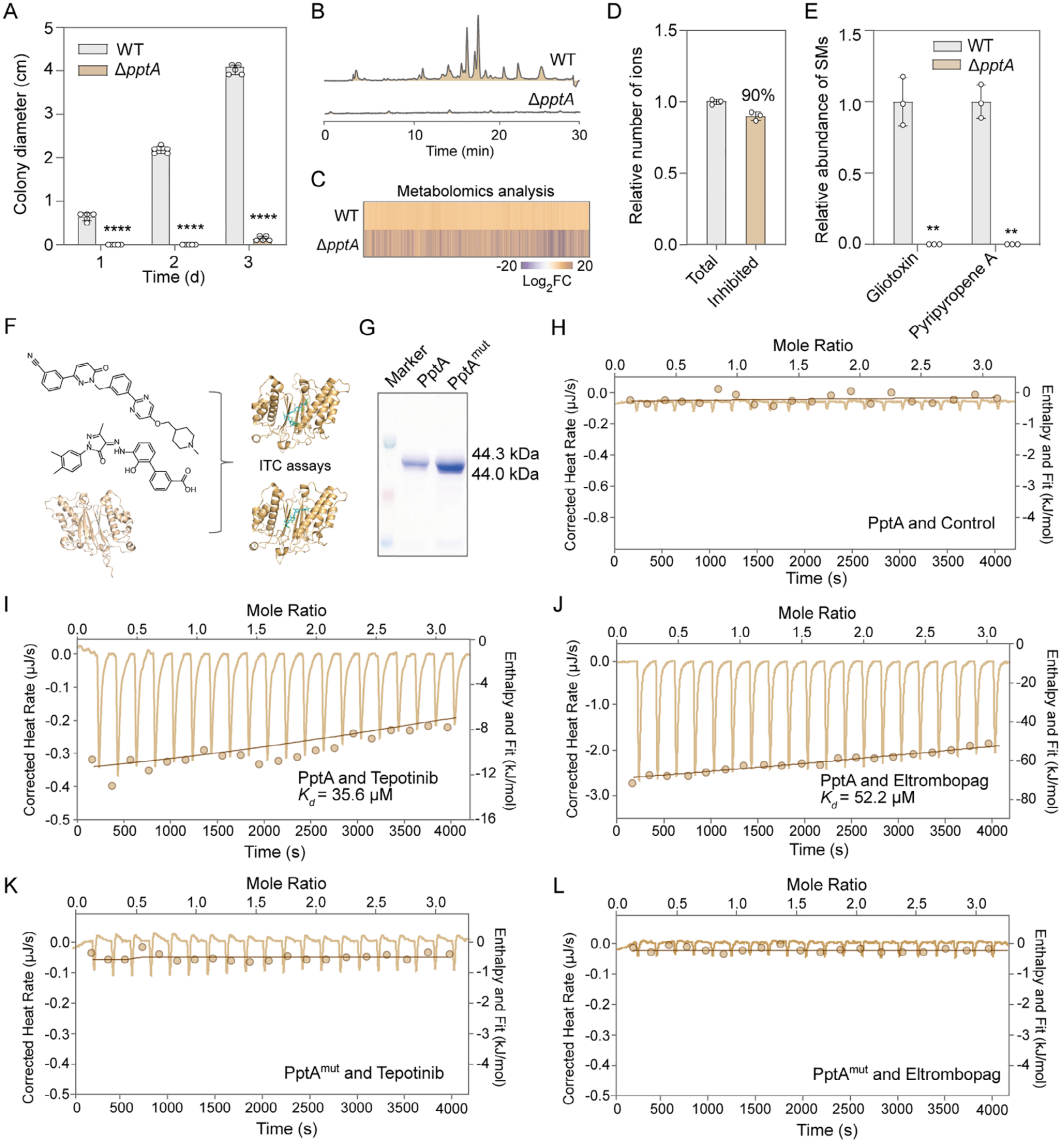
Function and identification of antifungal targets of tepotinib and eltrombopag. (A) Measurement of radial growth in *A. fumigatus* and its mutant strains (*n* = 5). (B) HPLC analysis of secondary metabolites in *A. fumigatus* and its mutant strains. UV absorptions at 254 nm are illustrated. (C) Heatmap shows the differences in metabolic ion abundances in *A. fumigatus* mutant vs. the control. (D) Metabolomic analysis of suppressed metabolic ion products in the *A. fumigatus* mutant. (E) Relative abundances of representative virulence‐associated secondary metabolites in the Δ*pptA* mutant and control strains. A Log_2_foldchange < −1 indicates repressed metabolic ions. (F) Structures and binding models of tepotinib, eltrombopag and PptA. (G) Brilliant blue G‐stained 12% SDS‐PAGE of PptA and PptA^mut^ proteins. Lane 1: Marker. Lane 2: His‐labeled PptA protein (44.3 kDa). Lane 3: PptA^mut^ (PptA with D159A, W227A, E231A, K235A mutations at the drug‐binding pocket), 44.0 kDa. (H–J) Isothermal titration calorimeter (ITC) assays showed the binding of PptA with PBS buffer (H), tepotinib (I), and eltrombopag (J). (K, L) ITC assays showed the binding of PptA^mut^ with tepotinib (K) and eltrombopag (L). All error bars are expressed as mean ± SD. Statistical analysis was performed using Two‐way ANOVA (Significant at ^**^ *p* < 0.01, ^****^ *p* < 0.0001).

To further investigate whether the candidate drugs act on PptA, the recombinant *His_6_*‐tagged PptA protein (44.3 kDa) was expressed and purified, and isothermal titration calorimetry (ITC) assays were performed (Figure 2F,G). Parallel control experiments were carried out by titrating PptA‐His into drug‐free buffer to exclude non‐specific interactions. ITC thermograms and fitted binding isotherms revealed an interaction between PptA and both tepotinib and eltrombopag, with thermodynamic constants (*K_d_*) of 35.6 and 52.2 µM, respectively (Figure 2H–J). These affinity values are consistent with the observed antifungal efficacy of the drugs. Moreover, alanine mutations targeting active‐site residues D159, W227, E231 and K235 of PptA were constructed to characterize their contributions to drug interaction. Following expression and purification of PptA^mut^ protein (44.0 kDa), ITC measurements against tepotinib and itepepeltin revealed an almost complete abolition of drug binding (Figure 2K,L). Collectively, both genetic and thermodynamic evidence support PptA as one of the functional targets of tepotinib and eltrombopag, providing a mechanistic basis for exploring therapeutic strategies targeting fungal metabolic pathways.

### 2.3 Antifungal Activity of Candidate Drugs Against a Broad Range of Clinical *A. fumigatus* Isolates

To assess the efficacy of tepotinib and eltrombopag against clinical isolates, an analysis was conducted on the PptA conservation and drug activity in *A. fumigatus* strains derived from patients with invasive aspergillosis (Figure 3A). Genomic data were gathered from 54 previously reported clinical isolates originating from Germany, Canada, and France, which included 30 azole‐sensitive strains, 19 azole‐resistant strains, and 5 isolates with unclassified susceptibility profiles [30] (Table S1). Conservation analysis of PptA indicated 100% coverage and 99% identity across all the examined clinical isolates, suggesting that this protein has not acquired resistance mutations under long‐term antifungal selection pressure (Figure 3B, Figure S13). Further examination of the drug‐binding sites confirmed that residues D159, W227, E231, and K235 remain strictly conserved among clinical strains (Figure 3C). Homology modeling of PptA (KAH1883280.1) from the azole‐resistant isolate *A. fumigatus* NRZ‐2016‐342, followed by structural alignment with PptA from the reference strain *A. fumigatus* A1163, yielded a root‐mean‐square deviation (RMSD) of 0.138, indicating high similarity between their three‐dimensional structures (Figure 3D, Figure S14). Molecular docking of both candidate drugs into PptA from NRZ‐2016‐342 replicated binding to the same key residues, implying a potential broad‐spectrum mechanism against diverse *A. fumigatus* isolates (Figure 3E).

**FIGURE 3:**
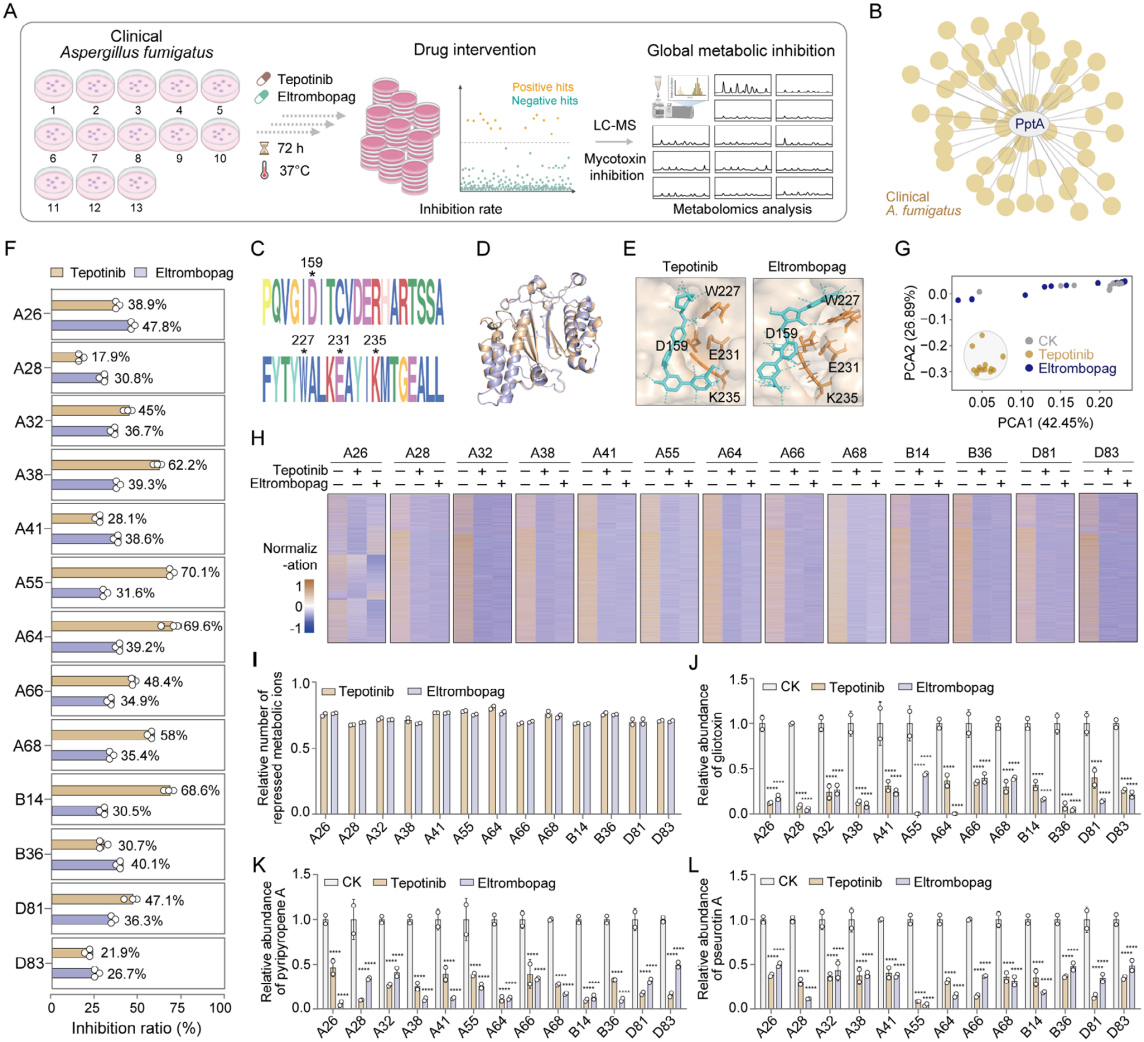
Broad‐spectrum activity of tepotinib and eltrombopag against clinical *A. fumigatus* isolates. (A) Workflow for drug activity evaluation assay. (B) Conservation analysis of PptA in 54 previously reported clinical strains. These strains were derived from Germany, Canada, and France, including 30 azole‐sensitive strains, 19 azole‐resistant strains, and 5 unclassified strains. (C) Conservation analysis of drug binding sites in PptA from clinical strains. (D, E) Conservation analysis of structure and drug binding sites in PptA from the clinical drug‐resistant *A. fumigatus* NRZ‐2016‐342. (F) Inhibition rates of tepotinib and eltrombopag against 13 collected clinical *A. fumigatus* strains. (G) PCA analysis of metabolomic data from clinical strains treated with tepotinib and eltrombopag. (H) Heatmap shows differences in metabolic ion abundances of clinical strains after treatment with two drugs. (I) Relative quantities of metabolic ions inhibited in clinical strains after treatment with two drugs. (J–L) Relative abundances of representative secondary metabolites, gliotoxin, pyripyropene A, and pseurotin A in clinical strains treated with two drugs. A Log_2_foldchange < −1 indicates repressed metabolic ions. All error bars are expressed as mean ± SD. Statistical analysis was performed using Two‐way ANOVA (Significant at ^****^ *p* < 0.0001).

Thirteen clinical isolates (from 10 patients with invasive pulmonary disease and 3 with chronic pulmonary disease) were collected, and the growth and metabolic inhibitory effects of the two candidate drugs at 200 µM against these strains were evaluated (Figure S15). Phenotypic analysis revealed that both drugs inhibited the growth of clinical isolates to different extents, with inhibition rates ranging from 17.9% to 70.1% for tepotinib and 26.7%–47.8% for eltrombopag (Figure 3F). Subsequent metabolic profiling of drug‐treated strains showed a marked reduction in secondary metabolite production (Table S5). Comparative metabolomics revealed highly similar metabolic ion profiles in the tepotinib‐treated group but divergent patterns in the eltrombopag group (Figure 3G, Figure S16A). Heatmap analysis demonstrated global suppression of metabolites in clinical isolates following drug treatment (Figure 3H). Inhibition rates ranged from 67.6% to 80.7% for tepotinib and 68.4%–76.8% for eltrombopag (Figure 3I). Specifically, tepotinib reduced the abundance of gliotoxin, fumiquinazoline C, pyripyropene A, and pseurotin A by 59.8%–100%, 51.7%–91.5%, 53.6%–89.9%, and 59.4%–91.2%, respectively; eltrombopag similarly decreased the levels of these four metabolites by 55.8%–100%, 54.7%–87.9%, 50.3%–93.5%, and 50.4%–95% (Figure 3J–L, Figure S16B,C). These results suggest that the candidate drugs exert a much stronger inhibitory effect on fungal metabolism than on growth. Collectively, these findings identify PptA as a conserved target in azole‐resistant and ‐susceptible *A. fumigatus*, implying tepotinib and eltrombopag could serve as broad‐spectrum antifungal candidates against invasive aspergillosis.

### 2.4 Broad‐Spectrum Antifungal Activity of Candidate Drugs Against Diverse Pathogenic Fungi

Given the conservation of the drug‐binding sites in PptA protein across the human pathogenic fungi *A. fumigatus*, *M. circinelloides*, *A. flavus*, *C. neoformans*, *C. gattii* and the plant pathogenic fungus *F. oxysporum* (Figure 1C), we hypothesized that tepotinib and eltrombopag exert broad‐spectrum antifungal activity. We modeled the three‐dimensional structures of PptA orthologs across diverse fungi using AlphaFold3 and performed structural alignments with the *A. fumigatus* PptA (Figure 4A,B). The RMSD values were 1.881, 0.335, 7.538, 7.984, and 1.104, respectively (Figure S17). These results indicate that PptA structures are highly consistent among filamentous fungi but diverge substantially in Basidiomycota. Further structural comparison of the active sites between *A. fumigatus* and other fungal PPTases revealed high structural consistency, with RMSD values of 0.304, 0.112, 0.367, 0.384, and 0.426, respectively (Figure S17).

**FIGURE 4:**
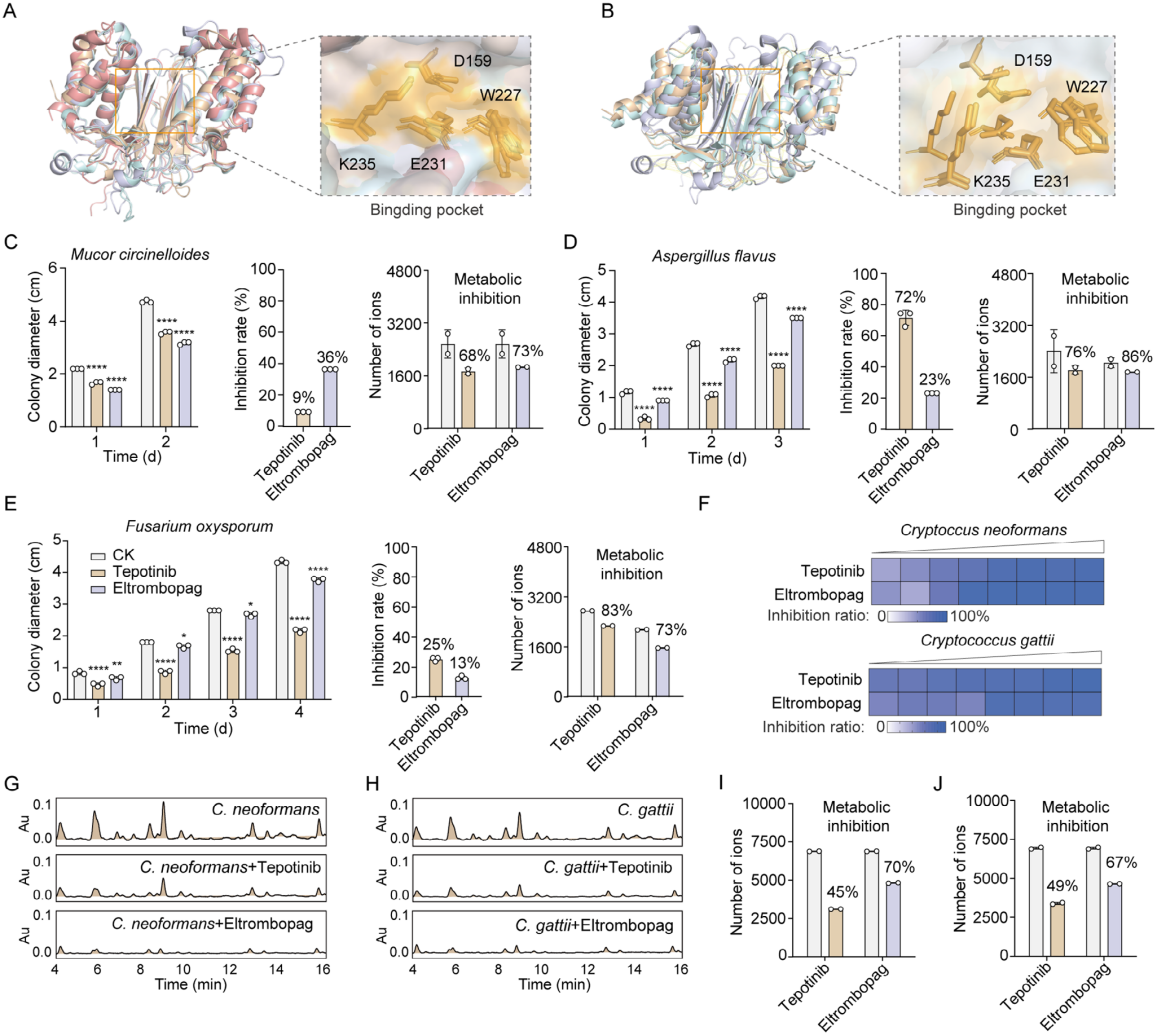
Broad‐spectrum inhibitory activity of tepotinib and eltrombopag on the metabolism of different pathogenic fungi. (A) Aligment analysis of structure and active pocket in PPTases across diverse filamentous fungi. Structural alignment of PptA from *A. fumigatus* with those from *M. circinelloides*, *A. flavus*, and *F. oxysporum* revealed RMSD values of 1.881, 0.335, and 1.104, respectively. (B) Conservation analysis of structure and active pocket in PPTases across diverse *Cryptococcus* species. Structural alignment of PptA from *A. fumigatus* with those from *C. neoformans* and *C. gattii* revealed RMSD values of 7.538 and 7.984, respectively. (C–E) Inhibitory activity of the two candidate drugs on the growth and metabolism of *M. circinelloides* (C), *A. flavus* (D), and *F. oxysporum* (E). (F) Inhibitory effects of two candidate drugs on the proliferation of *C. neoformans* and *C. gattii*. (G, H) HPLC analysis of metabolic profiles in *C. neoformans* (G) and *C. gattii* (H) treated with two candidate drugs. UV absorptions at 254 nm are illustrated. (I, J) Metabolomic analysis of the number of inhibited metabolic ions in *C. neoformans* (I) and *C. gattii* (J) following treatment with two candidate drugs. All error bars are expressed as mean ± SD. Statistical analysis was performed using Two‐way ANOVA (Significant at ^*^ *p* < 0.05, ^**^ *p* < 0.01, ^****^ *p* < 0.0001).

We next evaluated the antifungal activity of tepotinib and eltrombopag at 200 µM. Growth analysis of drug‐treated *M. circinelloides*, *A. flavus* and *F. oxysporum* showed inhibition rates of 9%, 72% and 25% for tepotinib, and 36%, 23% and 13% for eltrombopag (Figure 4C–E). To compare antifungal activity with the positive control amphotericin B (AmB), we determined the IC_50_ values of the two candidate agents against various fungal pathogens. The IC_50_ values of tepotinib against *M. circinelloides*, *A. flavus*, and *F. oxysporum* were 635.9, 116.6, and 867.5 µM, respectively. The corresponding IC_50_ values of eltrombopag were 181.9, 868, and 882.1 µM (Figure S18A). Notably, these values are substantially higher than the MIC values of AmB (0.45, 0.88, and 0.73 µM for the three fungi) (Figure S18B). Unexpectedly, total metabolic ion profiling showed that 3,386, 3,336 and 2,831 ions were detected in tepotinib‑treated *M. circinelloides*, *A. flavus* and *F. oxysporum*, respectively, while 3,176, 2,462, and 2,359 ions were found in the eltrombopag‑treated groups (Table S3). PCA and heatmap analyses of metabolic ion abundance revealed that drug treatment suppressed global metabolism (Figure S19). Comparative metabolomics revealed high metabolic inhibition rates, with values of 68%, 76% and 83% for tepotinib, and 73%, 86% and 73% for eltrombopag (Figure 4C–E). Moreover, both drugs inhibited proliferation and metabolism in *C. neoformans* and *C. gattii* (Figure 4F–H). The IC_50_ values of tepotinib were 3.23 and 0.70 µM, whereas those of eltrombopag were 2.56 and 2.39 µM. These values were higher than the IC_50_ values of the positive control AmB (0.24 and 0.32 µM) (Figure S18). Unexpectedly, metabolic inhibition rates of tepotinib reached 45% and 49%, while those of eltrombopag were 70% and 67%, respectively (Figure 4I,J, Figure S20). These data indicate that the drug‐binding pocket of PptA exhibits structural conservation among various fungal pathogens, which suggests the potential of tepotinib and eltrombopag as broad‐spectrum agents targeting fungal metabolism.

### 2.5 Combination Therapy of the Drug Candidates With Amphotericin B

Amphotericin B serves as a first‐line therapeutic agent for fungal infections; however, it is accompanied by significant side effects [31]. Considering that tepotinib and eltrombopag target different pathways from AmB, it was hypothesized that these two drug candidates exhibit synergistic effects with AmB, thereby reducing its dosage and enhancing its antifungal activity. Antifungal assays indicated that the inhibitory effect of AmB against *A. fumigatus* was enhanced in a concentration‐dependent manner. Complete suppression of growth was observed at a concentration of 12.8 µM (Figure 5A). Dose–response curve analysis revealed an IC_50_ value of 0.9227 µM for AmB (Figure S21). Accordingly, AmB was adopted as the positive control for the two drug candidates. In contrast, the IC_50_ values of tepotinib and eltrombopag were 100.4 and 765.9 µM, respectively (Figure 5B). Notably, when combined with tepotinib, complete growth inhibition of *A. fumigatus* was achieved with an AmB concentration of only 1.6 µM, while combination with eltrombopag achieved the same outcome at 6.4 µM (Figure 5C,D). Dose–response curves for the combinations demonstrated IC_50_ values of 0.1242 and 0.3541 µM for AmB in the presence of tepotinib and eltrombopag, respectively, with combination indices of 0.14 and 0.38 (Figure 5E).

**FIGURE 5:**
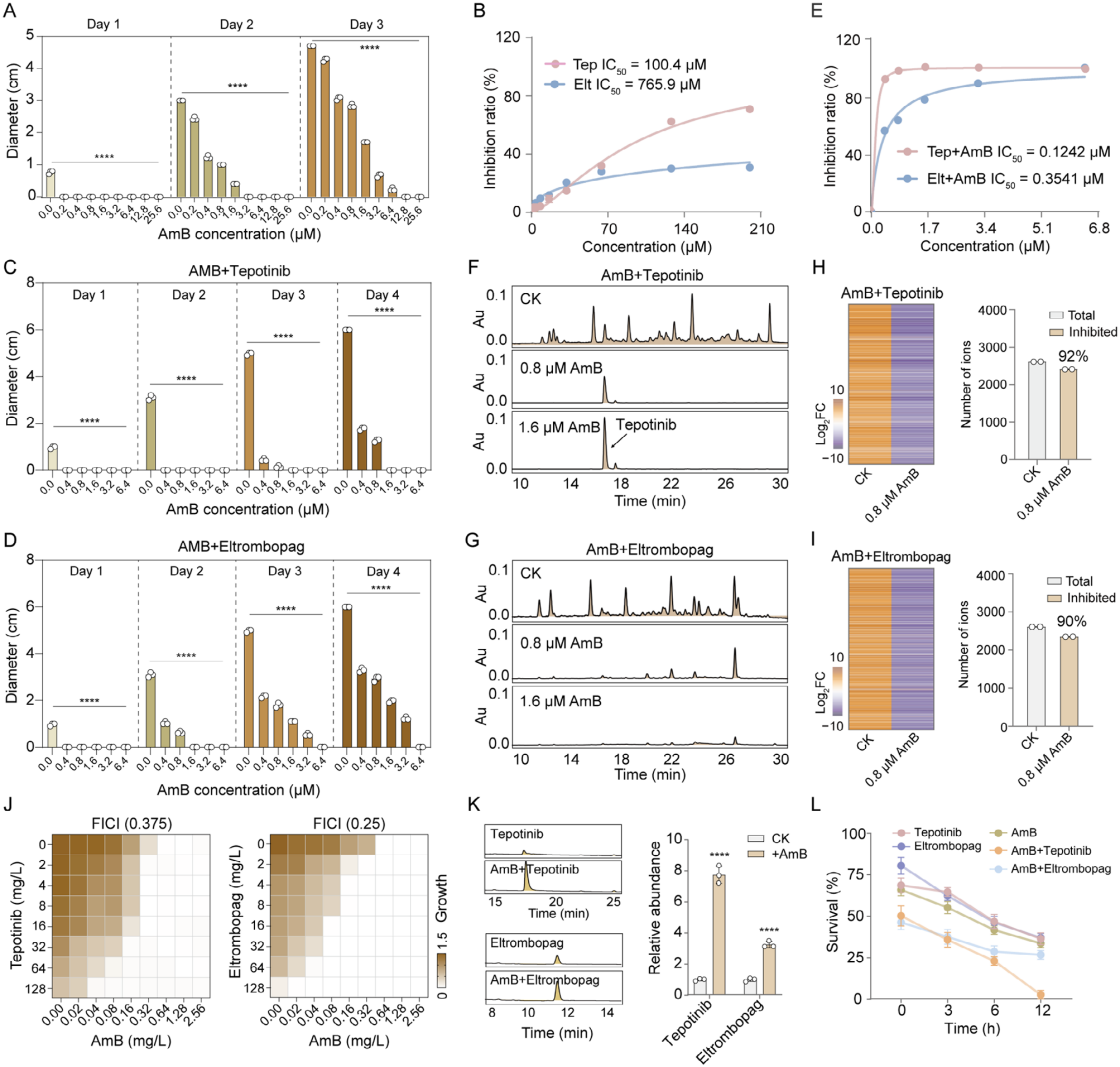
Synergistic antifungal activity of two candidate drugs in combination with amphotericin B. (A) Antifungal activity of amphotericin B (AmB) at different concentrations against *A. fumigatus*. (B) Evaluation of IC_50_ values of two candidate agents against *A. fumigatus*. (C, D) Antifungal activity of tepotinib (C) or eltrombopag (D) in combination with AmB against *A. fumigatus*. (E) Evaluation of IC_50_ values of tepotinib or eltrombopag in combination with AmB against *A. fumigatus*. (F, G) Evaluation of the metabolic inhibitory activity of tepotinib (F) or eltrombopag (G) in combination with AmB against *A. fumigatus*. (H, I) Metabolomic analysis of suppressed metabolic ions in *A. fumigatus* treated with tepotinib (H) or eltrombopag (I) in combination with AmB. (J) Fractional inhibitory concentration index (FICI) of tepotinib or eltrombopag combined with amphotericin B against *A. fumigatus* in RPMI 1640 medium. (K) HPLC analysis of intracellular drug accumulation in *A. fumigatus* treated with tepotinib or eltrombopag combined with amphotericin B at 230 nm. (L) Time‐kill curves of *A. fumigatus* treated with tepotinib or eltrombopag alone and in combination with amphotericin B. All error bars are expressed as mean ± SD. Statistical analysis was performed using Two‐way ANOVA (Significant at ^****^ *p* < 0.0001).

Metabolic profiling of *A. fumigatus* under combination treatment demonstrated near‐complete suppression of fungal metabolism at an AmB concentration of 0.8 µM (Figure 5F,G). A total of 2,608 metabolic ions were detected in the combination groups, and heatmap analysis confirmed global inhibition of fungal metabolism. Quantification analysis of differential metabolic ion abundances indicated that 92% and 90% of metabolism was repressed by the tepotinib–AmB and eltrombopag–AmB combinations, respectively (Figure 5H,I). Furthermore, checkerboard assays were carried out in RPMI 1640 medium to determine the fractional inhibitory concentration index (FICI) values for tepotinib or eltrombopag combined with AmB. The FICI values were 0.375 and 0.25, respectively; both were less than 0.5, indicating potent synergistic interactions (Figure 5J). Comparison of intracellular drug accumulation in *A. fumigatus* under monotherapy vs. combination treatment revealed that co‐treatment with AmB elevated intracellular levels of the two candidate compounds by 7.8‐fold and 3.3‐fold, respectively (Figure 5K). Consistently, time‐kill curves revealed significantly improved fungicidal activity in combination groups, verifying their synergistic fungicidal effects with AmB (Figure 5L). This dual‐target strategy, which simultaneously disrupts the metabolic pathway and cell membrane integrity, represents a novel paradigm shift in the combat of invasive fungal infection (Figure 6).

**FIGURE 6:**
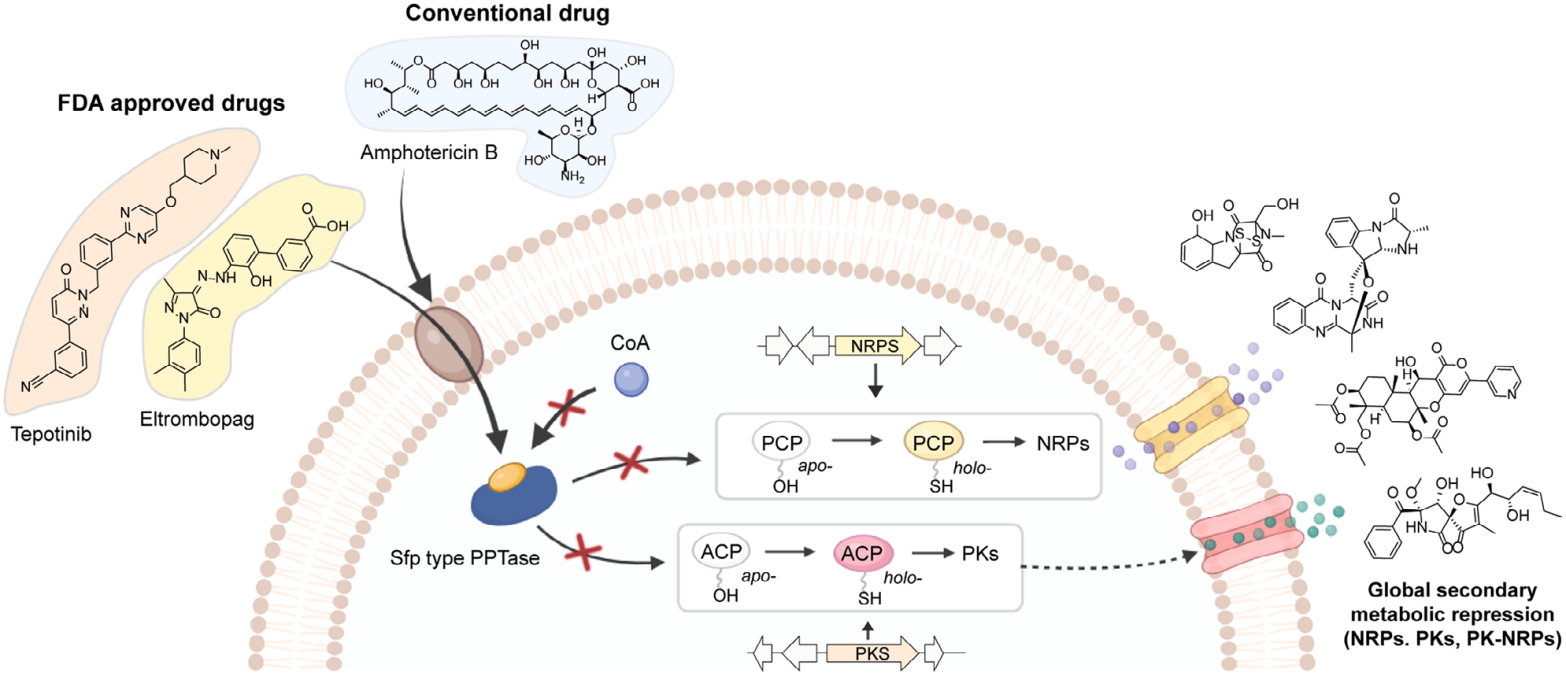
The model shows antifungal mechanisms of virulence abolition by tepotinib or eltrombopag in combination with amphotericin B. Upon combination antifungal therapy with tepotinib or eltrombopag plus amphotericin B, amphotericin B first binds ergosterol to form membrane pores, facilitating the intracellular entry of tepotinib/eltrombopag. These two agents bind to the active pocket of PPTase, block the biosynthesis of virulence‐related secondary metabolites, thereby exerting potent dual‑target antifungal effects to efficiently control fungal infections. NRPs: Nonribosomal peptides, PKs: Polyketides.

## 3 Discussion

In the context of rapid global environmental change and an increasing immunocompromised population, the extensive use of antifungal agents has spurred the emergence and evolution of drug resistance in human pathogenic fungi [6, 32, 33]. However, only a limited number of new antifungals have been approved in recent years, creating an urgent necessity for novel targets and strategies to expand therapeutic options and enhance the efficacy of existing agents [6, 9, 34]. This study introduces a novel anti‐infective strategy that targets the global secondary metabolic pathway in fungi. Through drug repurposing, two FDA‐approved drugs, tepotinib and eltrombopag, with broad‐spectrum antifungal activity, were identified. Both compounds decreased fungal colonization and expedited pathogen clearance in a murine model of invasive aspergillosis, and demonstrated synergistic effects with amphotericin B, which supports the development of combination therapies. Our findings offer a novel framework for antifungal intervention and present two promising candidate agents for the clinical treatment of invasive fungal infections.

Fungi utilize global secondary metabolism during infection to counter host defenses [14, 19, 35]. A detailed analysis of the enzymatic cascades in fungal secondary metabolite (SM) biosynthesis has identified PPTase (4’‐phosphopantetheinyl transferase) as an essential “molecular switch” for polyketide and non‐ribosomal peptide assembly [23, 26, 28]. The PPTase transfers 4’‐phosphopantetheine from coenzyme A to a conserved serine residue in peptidyl carrier proteins, activating them to drive downstream SM synthesis [25, 36]. Using this target, the three‐dimensional structure of PptA was predicted, and a high‐throughput screening of 3,019 FDA‐approved drugs identified tepotinib, an anticancer agent, and eltrombopag, a non‐peptide thrombopoietin receptor agonist, as novel antifungal compounds. Multiple factors account for their distinct targets in humans and fungi. Both agents possess polypharmacological chemical scaffolds with diverse pharmacophores that can interact with key residues in protein pockets. Fungal PptA has evolved binding cavity features, amino acid arrangements, and electrostatic surfaces suitable for accommodating these compounds. Comparable target switching has been widely reported for repurposed FDA drugs [37]. Furthermore, several challenges hinder the clinical application of tepotinib and eltrombopag as antifungals, such as modest in vitro potency, possible off‐target interactions, and uncertain long‐term safety.

The sequence and structural conservation of this target across both drug‐sensitive and drug‐resistant clinical isolates indicates strong negative selective pressure on its biological function during evolution, making mutational escape unlikely. This conservation endows target‐directed interventions with broad‐spectrum activity against diverse pathogenic fungi. In addition, PptA orchestrates fungal growth by governing lysine biosynthesis, which explains the dual activity of both candidate compounds—simultaneous suppression of growth and metabolism [28]. While our biochemical and genetic evidence firmly establishes PptA as the direct molecular target of tepotinib and eltrombopag, we cannot exclude the existence of additional fungal binding sites, which warrants dedicated follow‐up investigations. Collectively, these characteristics position this target as a promising candidate for combating difficult‐to‐treat fungal infections.

Drug repurposing and combination strategies can significantly shorten drug development timelines while reducing resistance and toxicity [38]. Although tepotinib and eltrombopag had higher IC_50_ values against *A. fumigatus* than amphotericin B, both notably decreased fungal burden and expedited pathogen clearance in a murine model of invasive aspergillosis. This disparity highlights their favorable in vivo efficacy, which is consistent with targeting fungal metabolism rather than essential lethal pathways, thus exerting weaker selection pressure and potentially delaying the evolution of resistance. Given the nephrotoxicity of amphotericin B, combination regimens are widely explored to lower its effective dose [39, 40]. In vitro co‐administration of the two candidates with amphotericin B, all FICI values were less than 0.5, confirming strong synergistic antifungal activity. Based on their distinct targets, we propose a mechanistic model: amphotericin B binds ergosterol and forms membrane pores, facilitating the intracellular entry of tepotinib/eltrombopag (Figure 6). These compounds bind to the active pocket of PPTase, inhibiting the biosynthesis of virulence‐associated SMs, thereby achieving effective infection control. Nevertheless, whether the interactions between the two candidate drugs and specific residues of PPTase hinder coenzyme A binding will be further validated via enzymatic inhibition assays in future investigations.

Unlike conventional antifungals that directly target essential genes, our strategy disarms fungi and significantly attenuates their pathogenicity: although fungi with reduced virulence remain viable, they lose the ability to colonize, invade, and evade immune clearance in the host, thus becoming susceptible to host immune elimination. This immune‐collaborative disarmament mode alleviates fungal suppression of host immunity and enables pathogen clearance even in immunocompromised hosts. Several important questions remain for future investigation: whether this strategy can achieve complete pathogen clearance in vivo, whether fungi may develop escape mechanisms against PPTase inhibition under prolonged drug pressure, and its long‐term in vivo safety profile. Addressing these questions will not only validate the potential of PPTase as a clinical antifungal target but also strengthen the rationale for metabolic pathway‐targeted antimicrobial strategies.

In summary, our research redefines antifungal therapy by redirecting the focus from the conventional targeting of the cell membrane to a metabolic bottleneck. This paradigm shifts from direct pathogen killing to attenuating fungal virulence, establishing metabolic inhibition as an alternative strategy to combat fungal infections. Collectively, we propose a metabolic targeting strategy and a therapeutic arsenal to address the growing threat of invasive fungal diseases.

## 4 Experimental Section

### 4.1 Mice

Balb/c mice were purchased from GemPharmatech Co., Ltd. (Nanjing, China). Male mice (age, 7–8 weeks) were injected with cyclophosphamide and cortisone acetate to generate a murine model of invasive aspergillosis.

### 4.2 Ethics Statement

All murine experiments were performed in strict accordance with the “Regulations of the Institute of Microbiology, Chinese Academy of Sciences of Research Ethics Committee.” The murine experiment protocol was approved by the Institute of Microbiology, Chinese Academy of Sciences of Research Ethics Committee (Permit No. APIMCAS2025273).

### 4.3 Strains and Cultivation

The strains used in this study are listed in Table S6. All *A. fumigatus* strains were grown at 37°C on glucose minimum medium (GMM) with appropriate supplements corresponding to the auxotrophic marker or antibiotics [41, 42]. *A. fumigatus* and its transformants were cultivated in liquid GMM medium at 25°C for 3 days to extract genomic DNA and for 5 days to detect secondary metabolites (SMs). *Escherichia coli* strains DH5α and BL21 were cultured in LB medium (1% tryptone, 0.5% yeast extract, 1% NaCl) supplemented with appropriate antibiotics to construct plasmids and express proteins, respectively.

### 4.4 Gene Deletion and Plasmid Construction

All plasmids and primers (BEIJING TSINGKE BIOTECH Co., Ltd., CHINA) are given in Table S6. For the construction of deletion mutants, around 1 kb upstream and downstream fragments of the targeted genes were amplified from *A. fumigatus* genomic DNA (gDNA) by high‐fidelity DNA polymerase TransStart *FastPfu* (TRANSGEN BIOTECH, CHINA). The marker gene *AfpyrG* was amplified from the vectors pYH‐WA‐pyrG and fused with the flanking sequences of the target gene to form different deletion cassettes by the double‐joint PCR method described previously [43]. The *pptA* gene in *A. fumigatus* was deleted according to the method described previously [44]. Briefly, the deletion cassette of *pptA* was transformed into *A. fumigatus* CEA17.2 to produce mutant TYSZL81. For protein expression, high‐fidelity DNA polymerase Q5 (NEW ENGLAND BIOLABS) was used to amplify the open reading frames (ORFs) of the targeted gene, which were then inserted into pET28a (*His_6_*‐tag) to produce pYHJ345 (PptA‐His) through the Clone Express MultiS One Step Cloning Kit (VAZYME BIOTECH Co. Ltd, CHINA). The pYSZL56 (PptA^mut^‐His) plasmids with D159A, W227A, E231A, and K235A substitutions were generated via an identical approach.

### 4.5 Biosynthetic Gene Cluster Analysis in Fungal Genomes

All fungal genomes (as of December 2025) were downloaded from the NCBI database, totaling 22 544 genomes. Using the ete3 toolkit, we queried the NCBI Taxonomy database to obtain the complete taxonomic lineage of each fungal genome based on its genus name, and completed the classification and statistics of the downloaded genomes. AntiSMASH 6.1.1 [45] was used to predict secondary metabolite biosynthetic gene clusters (BGCs) in all fungal genomes, and the proportion of each type of BGC was statistically analyzed.

### 4.6 Phylogenetic Analysis of Sfp‐Type PPTases

The PptA (EDP54396.1) in *A. fumigatus* was used as the query for a BLAST analysis at the NCBI website (www.blast.ncbi.nlm.nih.gov/Blast.cgi). Amino acid sequences of PptA homologues from 302 species were downloaded from the NCBI database, aligned with MEGA7 software, and manually adjusted. The phylogenetic trees were constructed using MEGA7 software, and clustering was performed by the neighbor‐joining method, while the other parameters were default. The species from plants were regarded as the outgroup of the Sfp‐type PPTases phylogram. The reliability of internal branches was evaluated with 1,000 bootstrap resamplings. The phylogram was modified and optimized via the Interactive Tree of Life website (ITOL, http://itol.embl.de/).

### 4.7 Structure Prediction and Virtual Screening

To screen antifungal drugs, the three‐dimensional structure of PptA containing 357 amino acids was predicted by AlphaFold3 [46]. The resulting model achieved an overall pLDDT score of 91, demonstrating far higher structural reliability compared with models from other prediction tools. BatchVinaGUI provides a streamlined graphical interface built on AutoDock Vina's robust scoring function, enabling high‐throughput batch molecular docking for dozens of ligands simultaneously, and supports unified configuration of docking parameters across all compounds. BatchVinaGUI screened 3,019 small molecules from the FDA‐approved drug library. RDKit converted their 2D structures to 3D conformations, leaving 1,787 dock‐qualified compounds that were converted to PDBQT format at pH 7.4. Semi‐flexible docking was conducted. The box, centered at the key residues’ geometric centroid (*x* = 5.453, *y* = 2.106, *z* = 1.8), had dimensions 4.5‐fold the molecular gyration radius, exhaustiveness = 16. A total of 1,280 ligands matched successfully [47, 48]. The ligands were ranked according to the binding affinity of PptA with different small molecule configurations (Table S2). Topological models and binding sites were visualized by the PyMOL2.5 software package. The default parameters of the tools were used.

### 4.8 Broad‐Spectrum Activity Determination of Drug Candidates

To evaluate the broad‐spectrum activity of tepotinib (T6121) and eltrombopag (T2562, TargetMol, USA), the human pathogens *A. fumigatus*, *A. flavus*, *M. circinelloides*, *C. neoformans*, *C. gattii*, and the plant pathogen *F. oxysporum* were all tested. The 1,000 spores of *A. fumigatus* and *A. flavus* were incubated in GMM medium containing 200 µM drugs, and the inhibition ratios were calculated after 3–4 days, and the metabolic ion products were analysed after 4–5 days of dark culture at 37°C. The mycelia of *M. circinelloides* and *F. oxysporum* were incubated in PDA medium containing 200 µM drugs, and the inhibition ratios were calculated after 2 days or 4 days, and the metabolic ion products were analysed after 3 days or 5 days of dark culture at 28°C. The *C. neoformans* and *C. gattii* were cultured in YPD medium to OD_600_ = 0.1 at 30°C with shaking at 220 rpm, then diluted 1000 times and incubated with drugs in a 96‐well plate. The inhibition ratio was calculated after 1 day, and the metabolic ionic products were detected after 2 days of incubation. The inhibition ratio of clinical strains was measured by the same method. To determine IC_50_ values of the two candidates against various fungi and compare their potency with amphotericin B (positive control), all strains were treated with the candidate drugs at 0, 2, 4, 8, 16, 32, 64, 128 and 200 µM. Amphotericin B was applied at 0, 0.2, 0.4, 0.8, 1.6, 3.2, 6.4, 12.8 and 25.6 µM for filamentous fungi, and 0, 0.1, 0.2, 0.3, 0.4, 0.5, 0.6, 0.7 and 0.8 µM for *Cryptococcus* species. IC_50_ values were calculated for each compound against individual fungi to compare antifungal activity. At least two replicates were performed for each strain.

### 4.9 HPLC and Metabolomics Profiling of Fungal Secondary Metabolites

After drug treatment, the cultures were extracted with 10 mL of ethyl acetate and evaporated under reduced pressure. The extracts were dissolved in 1 mL methanol (MeOH), and then analysed by HPLC equipped with an ODS column (C18, 250 × 4.6 mm, Waters XTERRA, 5 µm) with a flow rate of 1 mL/min. MeOH and water with 0.1% (v/v) formic acid were used as the elution solvent, and linear gradient conditions were as follows: 10%‐100% MeOH in 0–30 min, 100% MeOH in 30–35 min, and 10% MeOH in 35.1–40 min. Intracellular tepotinib in *A. fumigatus* was analyzed using the same protocol. For eltrombopag, the linear gradient consisted of 60%–95% MeOH (0–5 min), 95%–100% MeOH (25 min), and 60% MeOH (30.1–35 min). Other conditions were unchanged, and drug accumulation was monitored at 230 nm. Metabolomic analysis was performed by LC‐MS equipped with an Agilent 1200 LC/MSD SL (Santa‐Clara, USA) with a flow rate of 1 mL/min. The acetonitrile and water with 0.1% (v/v) formic acid were used as the elution solvent, and linear gradient conditions were as follows: 5%‐100% acetonitrile in 0–35 min, 100% acetonitrile in 35–40 min, 100%–5% acetonitrile in 40–40.1 min, and 5% acetonitrile in 40.1–45 min. The metabolomics of the drug‐treated strains were analysed by the same method. The mass spectrum data were collected and converted into a format containing retention time, *m/z*, and ion peak density, respectively. Repressed metabolites were screened with |log_2_foldchange < ‐1 as the threshold.

### 4.10 Animal Model of *A. fumigatus* Infection

The therapeutic effects of tepotinib and eltrombopag were evaluated in a murine invasive aspergillosis model. Briefly, male Balb/c mice (GemPharmatech (Nanjing, China)) weighing about 19–21 g were immunosuppressed via administration of cyclophosphamide by separate intraperitoneal injections, one at 3 days (200 mg·kg^−1^ of body weight) and the other at 1 day (200 mg·kg^−1^ of body weight) before infection. The second treatment includes administration of cortisone acetate at a dose of 250 mg·kg^−1^ by separate subcutaneous injection at 1 day before infection. Anesthetized mice (10 mice/fungal strain) were infected by nasal instillation of 20 µL of 1 × 10^8^ conidia/mL (day 0) and monitored three times daily. Given the well‐tolerated murine dosage windows of 6–15 mg/kg for tepotinib and 17.6–50 mg/kg for eltrombopag, infected mice received intranasal treatment at 10.0 mg/kg on days 1, 3 and 5 post‐infection (*n* = 10 per group), with vehicle controls administered an equal volume of PBS. Lungs were harvested from each group on day 6 post‐infection for fungal burden and histopathological analysis.

### 4.11 Fungal Burden and Histopathological Analysis

Lung tissues following drug treatment were removed and freeze‐dried. Subsequently, the dry lung tissue was homogenized in a CTAB (Cetyl trimethylammonium bromide, Sigma) extraction buffer (100 mM Tris‐HCl pH 7.5, 0.7 M NaCl, 10 mM EDTA, 1% CTAB, 1% β‐mercaptoethanol) to extract total gDNA as described previously [49]. Briefly, mixed samples were lysed at 65°C for 30 min, mixed with 1 mL chloroform, and centrifuged at 1500 ×g for 10 min. Supernatants were mixed with an equal volume of isopropanol, centrifuged at 1500 ×g for 10 min, and the resulting precipitate was washed with 70% ethanol and resuspended in distilled water. A standard curve was generated by twofold serial dilution of *A. fumigatus* genomic DNA (gDNA) from 1 ng/µL to yield 12 concentrations, with the log_2_ of known standard concentrations as the X‐axis and corresponding *Ct* values as the Y‐axis. Extracted gDNA samples were diluted to 20 ng/µL, and qPCR quantification was performed using *Afks1* as the internal reference gene. The abundance of *A. fumigatus* in lung tissues was calculated from the standard curve.

Lungs removed from mice infected for 6 days were fixed with 10% formalin and subsequently embedded in paraffin. Subsequently, consecutive slices with 4–6 µm in thickness were obtained and stained with haematoxylin‐eosin (HE) and periodic acid‐schiff (PAS) for histopathological studies.

### 4.12 Expression and Purification of Proteins

*E. coli* BL21 cells transformed with pYHJ345 or pYSZL56 were incubated at 37°C in LB medium containing 50 µg·mL^−1^ kanamycin until OD_600_ = 0.6 to express PptA‐His or PptA^mut^‐His recombinant protein. Next, 0.5 mM isopropyl β‐D‐1‐thiogalactopyranoside (IPTG) was used to induce protein expression at 16°C for 20 h. The cells collected by centrifugation were lysed (50 mM NaH_2_PO_4_·2H_2_O, 300 mM NaCl, 10 mM imidazole, pH 8) by freeze‐thaw, and debris was removed. The supernatant was subjected to purification using Ni‐NTA resin (QIAGEN, CA) with wash buffer (40 mM imidazole) and elution buffer (100 mM imidazole). Target proteins were detected by 12% SDS‐PAGE and quantified using a Nano‐Drop C2000 (Thermo Fisher Scientific).

### 4.13 Isothermal Titration Calorimetry (ITC) Assays

To measure the binding affinity and thermodynamics between PptA and tepotinib/eltrombopag, all samples were dissolved in PBS buffer to ensure consistent experimental conditions. PptA or PptA^mut^ protein was concentrated to 500 µM as the titrant, while each drug was diluted to a final concentration of 50 µM as the analyte. Prior to measurement, all samples were degassed under vacuum for 10 min to remove air bubbles, followed by 20 consecutive titrations at 25°C. Data were analyzed strictly in accordance with the previous method [50] using Launch Nano Analyze software (version 3.11.0). For the control group, the same concentration of PptA protein was titrated into PBS buffer to exclude the heat generated by non‐specific binding. Experiments were independently repeated twice.

### 4.14 Evaluation of the Combined Antifungal Efficiency Between Candidate Drugs and Amphotericin B

Amphotericin B was prepared into a solution with a concentration of 20 mM and then successively diluted into GMM plates with final concentrations of 0, 0.2, 0.4, 0.8, 1.6, 3.2, 6.4, 12.8, 25.6, 51.2, 100, and 200 µM, respectively. The 1000 spores of *A. fumigatus* were point‐incubated in GMM medium and cultured at 37°C for 3 days without light. The colony diameter was measured daily, and the inhibition ratio for 3 days was calculated. Subsequently, 200 µM tepotinib was mixed with 0, 0.4, 0.8, 1.6, 3.2, and 6.4 µM amphotericin B to form GMM medium. *A. fumigatus* was cultured in GMM medium for 3 days to calculate the inhibition ratio, and the secondary metabolites were analysed after 4 days. The antifungal efficacy evaluation of eltrombopag combined with amphotericin B was the same as above. Checkerboard assays in 96‐well plates with RPMI 1640 medium were performed to assess synergism between varying concentrations of each candidate compound and amphotericin B. Absorbance at OD_600_ was measured after 48 h for FICI calculation. For time‐kill analysis, fungal spores were incubated with 0.3 µM amphotericin B, 50 µM each candidate, or their combinations in RPMI 1640 medium. Viable spore counts were determined at 0, 3, 6 and 12 h to generate time‐kill curves. HPLC was used at 24 h to quantify intracellular drug accumulation, further characterizing the combinatorial interaction. Two replicates were performed for each strain.

### 4.15 Quantification and Statistical Analysis

Statistical parameters are shown in the corresponding Figure legends. All statistical analyses were done in GraphPad Prism8 software. Quantification data are generally presented as bar/line plots, with the error bar representing mean ± SD. Asterisks were used to indicate statistical significance, ^*^stands for *p* < 0.05; ^**^ *p* < 0.01, ^***^ *p* < 0.001, and ^****^ *p* < 0.0001.

## Author Contributions

W.‐B.Y. performed the conceptualization, supervision, and funding acquisition. W.‐B.Y. and Z.S. contributed to all stages of manuscript preparation and editing. Z.S. performed fungal phenotypic analysis, HPLC analysis, metabolome analysis, phylogenetic analysis, structural prediction, molecular docking, ITC assays, and murine experiments. C.Z. analyzed and evaluated the data. Q.W. performed fungal SMs extraction. H.Z. performed the protein expression and purification. X.L., L.W., and M.B. participated in the discussion of the results. Z.S. and W.‐B.Y. wrote the paper. All authors have seen and approved the manuscript.

## Funding

This work was supported by the Chinese Academy of Sciences Project for Young Scientists in Basic Research [grant no. YSBR‐111], the Strategic Priority Research Program of Chinese Academy of Sciences [grant no. XDB0830000], and the National Natural Science Foundation of China [grant no. 32470046].

## Conflicts of Interest

The authors declare no conflicts of interest.

## Supporting information




**Supporting File 1**: advs77354‐sup‐0001‐SuppMat.pdf.

**Supporting File 2**: advs77354‐sup‐0002‐SuppMat.zip.

## Data Availability

The data that supports the findings of this study are available in the supplementary material of this article.
