# Supplementary material for: Repurposing FDA‐Approved Drugs to Inhibit Fungal PPTases for Broad‐Spectrum Synergy
Source: Adv Sci (Weinh). 2026 Aug 21;13(63):e77354. doi: 10.1002/advs.77354 (PMC13496288; doi:10.1002/advs.77354)
Supplement: Supplementary file 1 [file ADVS-13-e77354-s001.pdf]

Supporting Information

Repurposing FDA-Approved Drugs to Inhibit Fungal PPTases for Broad-Spectrum Synergy

*Zili Song, Can Zhao, Qiaoling Wang, Hongjiao Zhang, Xiao Liu, Linqi Wang, Michael Bromley, Wen-Bing Yin**

*Corresponding author: yinwb@im.ac.cn

This file includes:

Figure S1: Distribution of biosynthetic gene clusters (BGCs) in fungi.
Figure S2: Target of drug candidates in the biosynthetic pathway of fumiquinazoline C.
Figure S3: Phylogenetic analysis of the sfp-type PPTases.
Figure S4: Conservative analysis of amino acid sequences in fungal sfp-type PPTases.
Figure S5: Prediction of PptA protein structure and molecular docking with small-molecule drug ligands.
Figure S6: Structure and clinical trial information of the top 8 drugs with high affinity to PptA.
Figure S7: Interaction models of the top 8 FDA-approved drugs with high affinity to PptA.
Figure S8: Binding sites analysis of two drug candidates with antifungal activity in PptA.
Figure S9: Phenotype and metabolite analysis of *A. fumigatus* CEA17 treated with the top 8 drugs.
Figure S10: Metabolomic analysis of *A. fumigatus* CEA17 treated with tepotinib and eltrombopag.
Figure S11: Periodic Acid-Schiff staining of *A. fumigatus* in the mouse lungs after 5 days of treatment with two drug candidates.
Figure S12: Construction and metabolomics analysis of the Δ*pptA* mutant in *A. fumigatus*.
Figure S13: Phylogenetic analysis of the sfp-type PPTases in clinical *A. fumigatus*.
Figure S14: Conservation of PptA structure and drug-binding sites in clinical drug-resistant strain.
Figure S15: Phenotypic and HPLC analysis of clinical strains treated with 2 drug candidates.
Figure S16: Metabolomic analysis of clinical strains treated with 2 drug candidates.
Figure S17: Structural conservation of PPTases across diverse pathogenic fungi.
Figure S18: Evaluation of IC_{50} values of two candidate compounds and the positive control AmB against diverse pathogenic fungi.
Figure S19: Metabolomic analysis of diverse filamentous fungi treated with tepotinib and eltrombopag.
Figure S20: Metabolomic analysis of *Cryptococcus* species treated with tepotinib and eltrombopag.
Figure S21: The activity of positive control amphotericin B against *A. fumigatus*.
Legends for Supplementary Table S1 to S6

Additional files:

Supplementary Table S1 to S6.

Supplementary Figures

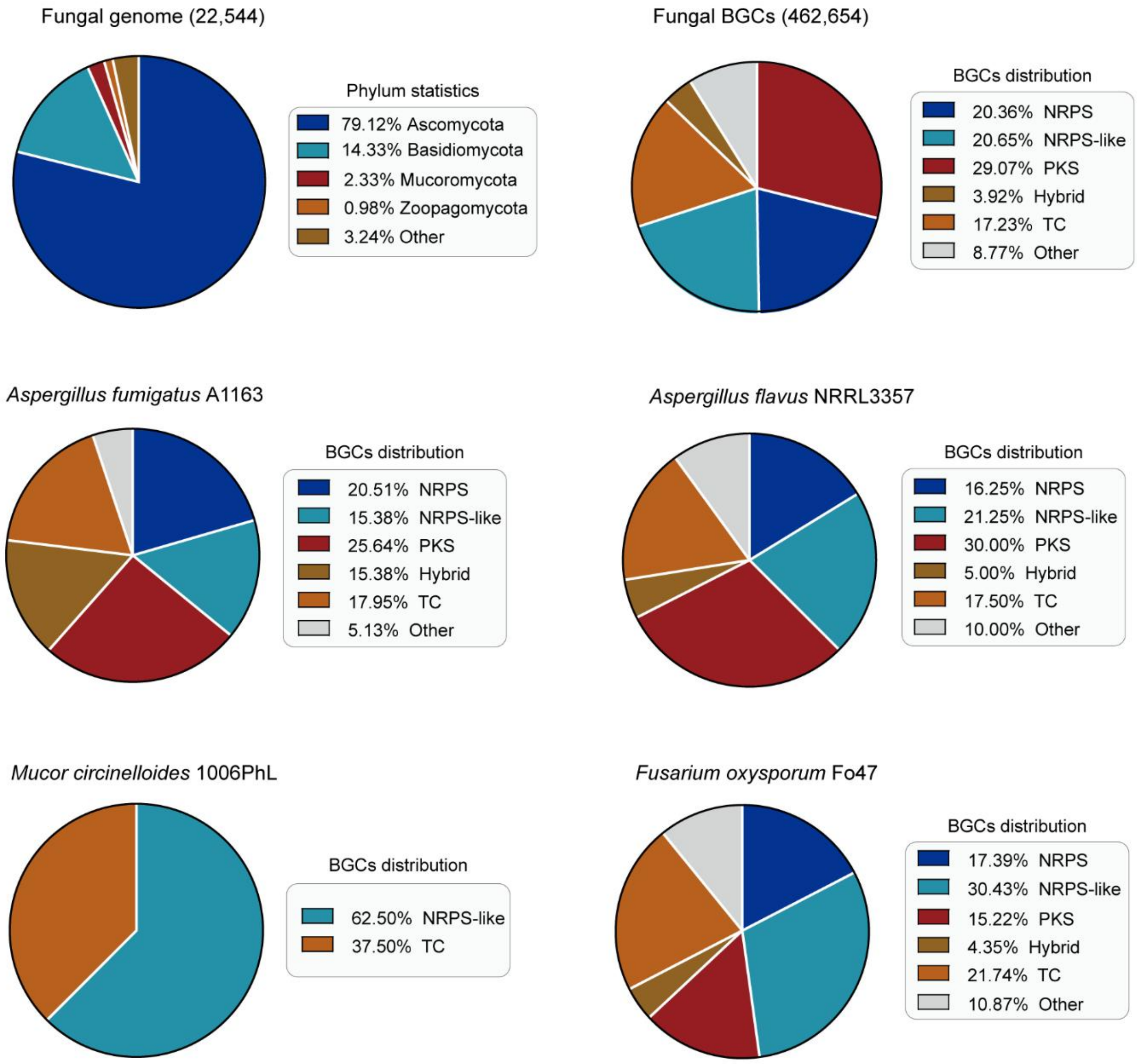

Figure S1. Distribution of biosynthetic gene clusters (BGCs) in fungi. Based on differences in the biosynthetic pathways of secondary metabolites, they are classified into nonribosomal peptide synthase (NRPS), NRPS-like, polyketide synthase (PKS), hybrid synthases (containing at least one of PKS or NRPS), terpene cyclase (TC) and other.

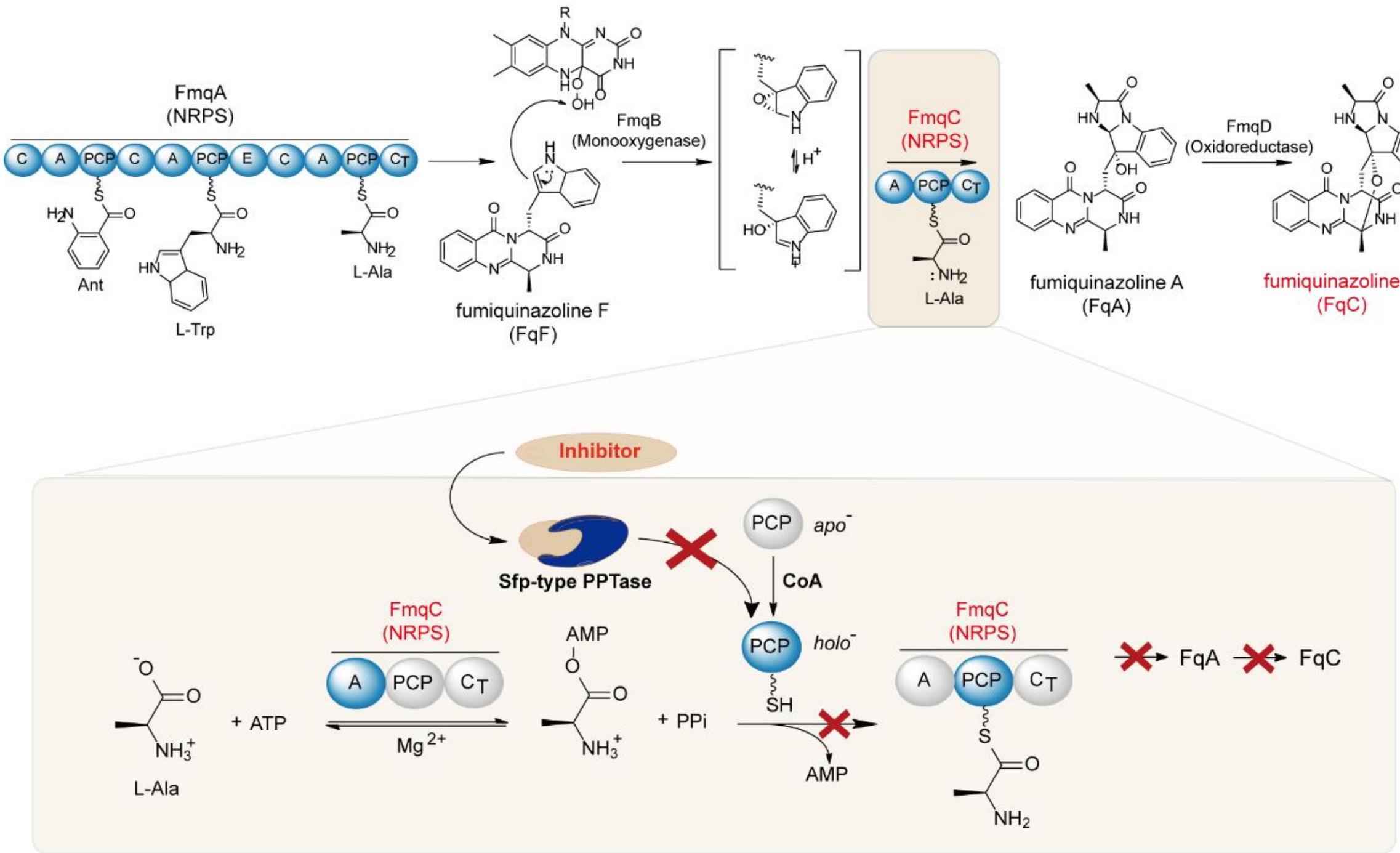

Figure S2. Target of drug candidates in the biosynthetic pathway of fumiquinazoline C. The biosynthesis of fumiquinazoline C depends on the sfp-type 4'-phosphopantetheinyl transferases (PPTases) PptA-catalyzed transfer of phosphopantetheine group from coenzyme A (CoA) to the conserved serine residue of carrier protein, which changes the carrier protein from inactive *apo*- form to active *holo*- form.

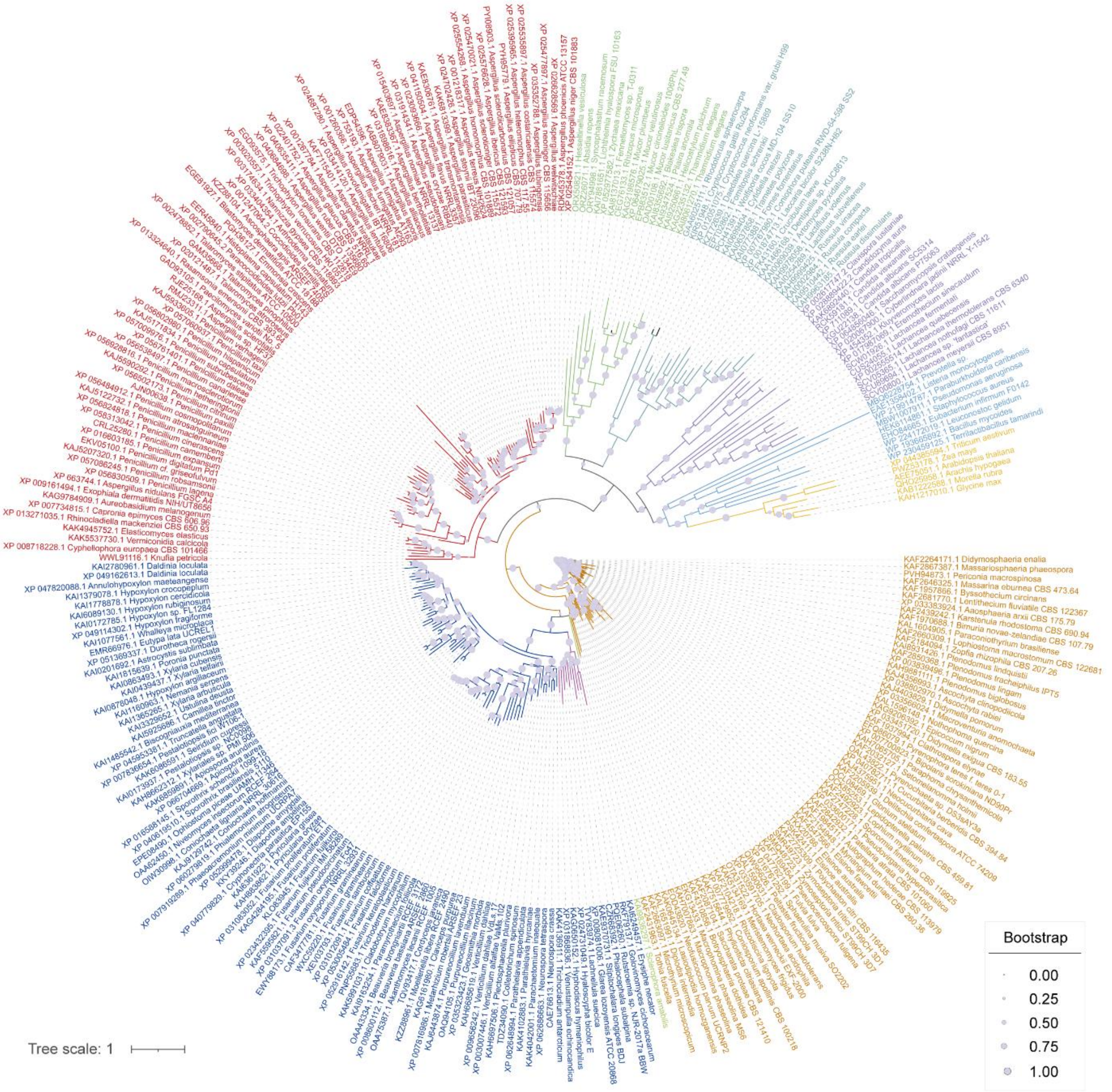

Figure S3. Phylogenetic analysis of the sfp-type PPTases. Different colors represent taxonomic units of different level. Six plant-derived sfp-type PPTases were considered as the outgroups.

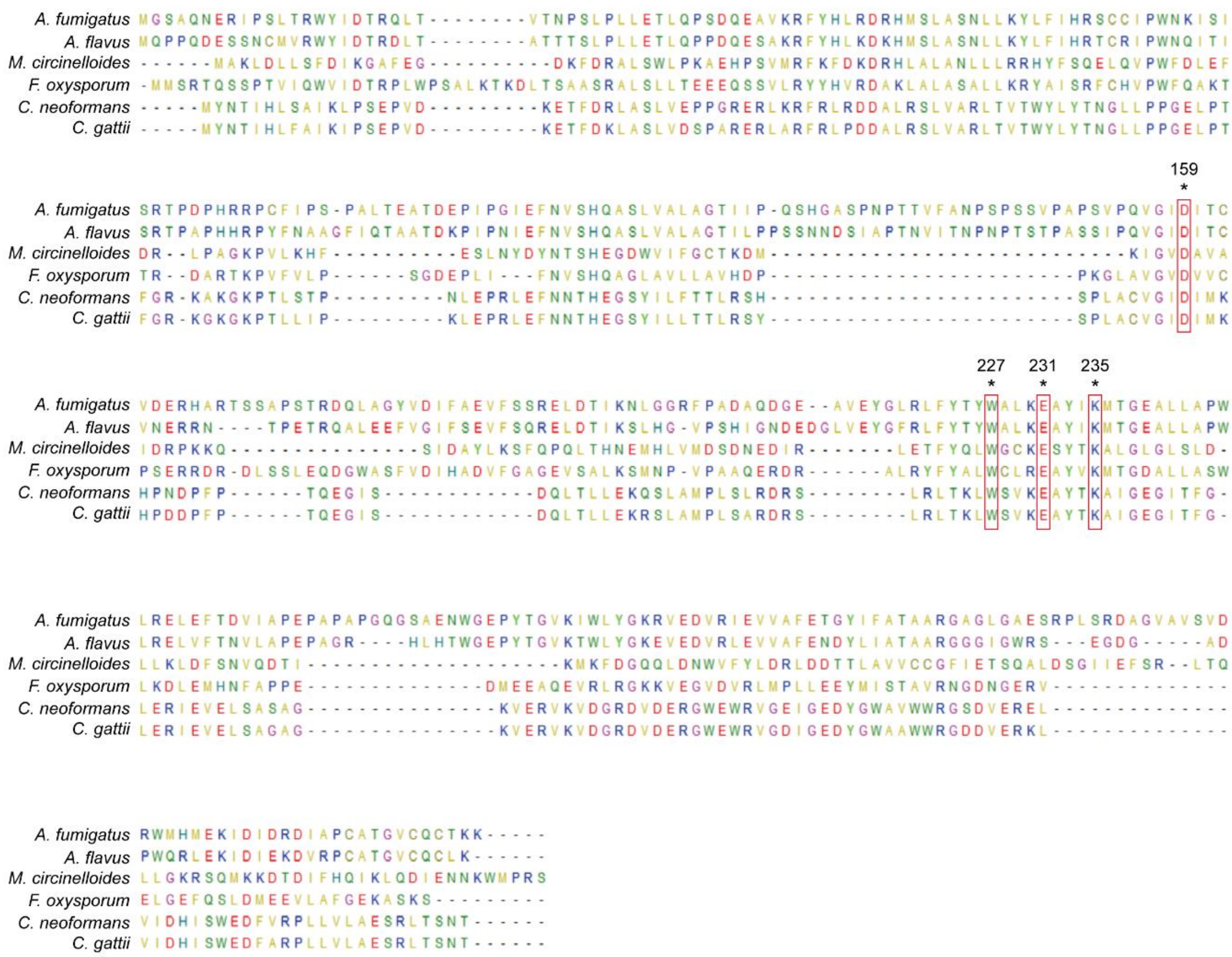

Figure S4. Conservative analysis of amino acid sequences in fungal sfp-type PPTases. The D159, W227, E231, and K235 were conserved in fungal sfp-type PPTases, including *Aspergillus fumigatus*, *A. flavus*, *Mucor circinelloides*, *Fusarium oxysporum*, *Cryptococcus neoformans*, and *C. gattii*.

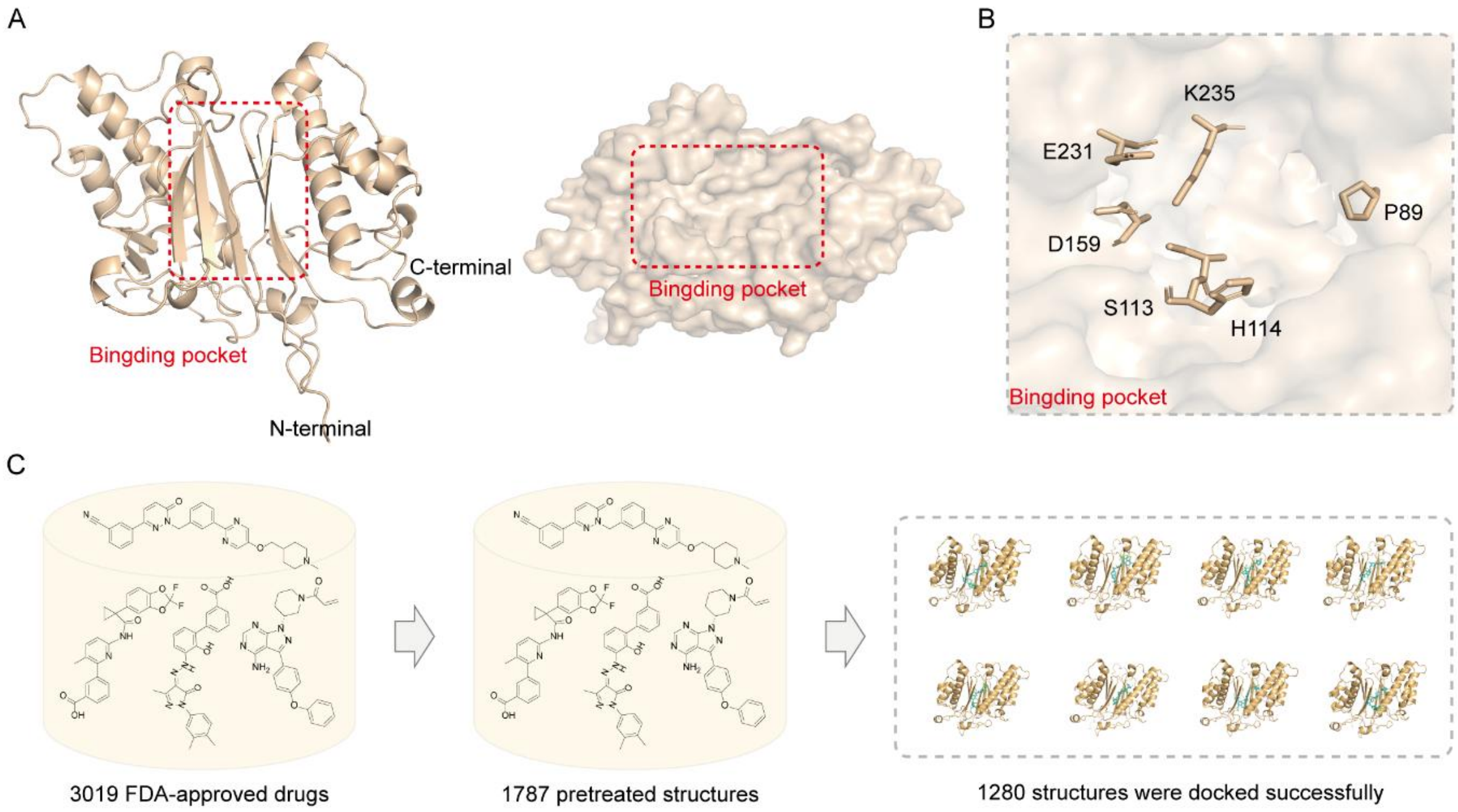

Figure S5. Prediction of PptA protein structure and molecular docking with small-molecule drug ligands. **A**) The predicted three-dimensional structure of PptA protein obtained by AlphaFold3. *N*-terminal, the starting site for protein synthesis. *C*-terminal, the termination site of protein synthesis. **B**) Key amino acid residues in the binding pocket of PptA protein. **C**) Preprocessing and molecular docking of FDA-approved small-molecule drug ligands.

A

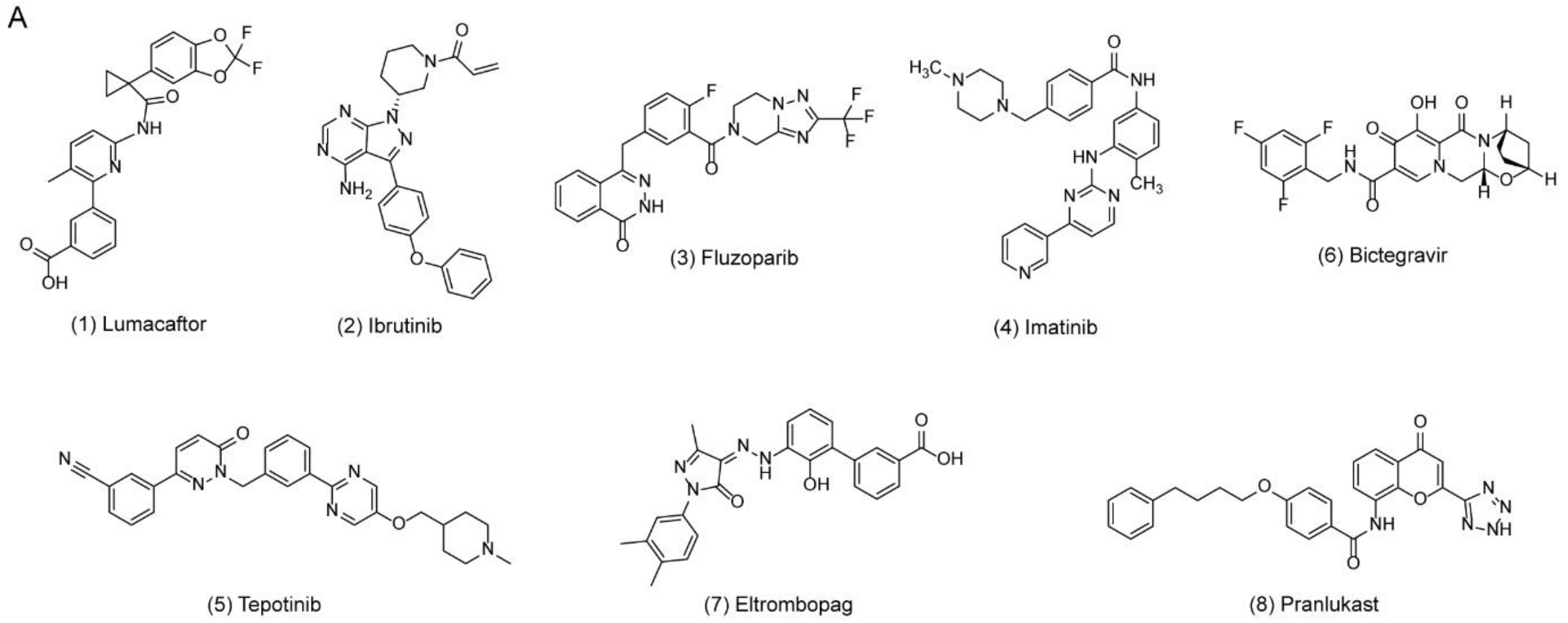

B

FDA drugs	Clinical trial	Biological activity
(1) Lumacaftor	Cystic Fibrosis, Phase 1, June 2013<br>Long QT Syndrome, Phase 2, June 15, 2021<br>Cystic Fibrosis|Advanced Lung Disease, Phase 3, March 2015	CFTR modulator
(2) Ibrutinib	Leukemia, Phase 2, February 27, 2012<br>Chronic Lymphocytic Leukemia, Phase 1, March 9, 2018<br>Small Lymphocytic Leukemia (SLL)|Chronic Lymphocytic Leukemia (CLL), Phase 2, July 31, 2020	Irreversible Btk inhibitor
(3) Fluzoparib	Advanced solid tumor, Phase 1, September 7, 2019<br>NSCLC|Non-small Cell Lung Cancer, Phase 2, July 2023<br>Ovarian Cancer, Phase 2, December 24, 2021	An orally active PARP1 inhibitor, superior antitumor activity
(4) Imatinib	Gastrointestinal Stromal Tumors, Phase 1, December 1, 2021<br>Leukemia, Phase 2, April 2001<br>Kidney Cancer, Phase 2, January 2006	An orally bioavailable tyrosine kinases inhibitor
(5) Tepotinib	FDA approves tepotinib for metastatic non-small cell lung cancer, February 15, 2024	An orally active and highly selective, reversible, ATP-competitive c-Met inhibitor
(6) Bictegravir	HIV-1 Infection, Phase 1, October 24, 2014	A potent inhibitor of HIV-1 integrase
(7) Eltrombopag	Myelodysplastic Syndrome (MDS)|Thrombocytopenia, Phase 1, April 6, 2011<br>Hepatitis C, Phase 1, January 19, 2009	An orally active thrombopoietin receptor nonpeptide agonist
(8) Pranlukast	Chronic Sinusitis, Phase 3, December 2006<br>Rhinitis, Allergic, Season, Phase 3, November 2004	A highly potent, selective and competitive antagonist of peptide leukotrienes

Figure S6. Structure and clinical trial information of the top 8 drugs with high affinity to PptA. **A**) Structures of top 8 FDA-approved drug with high affinity for PptA**. B**) Clinical trial information for the top 8 FDA-approved drugs.

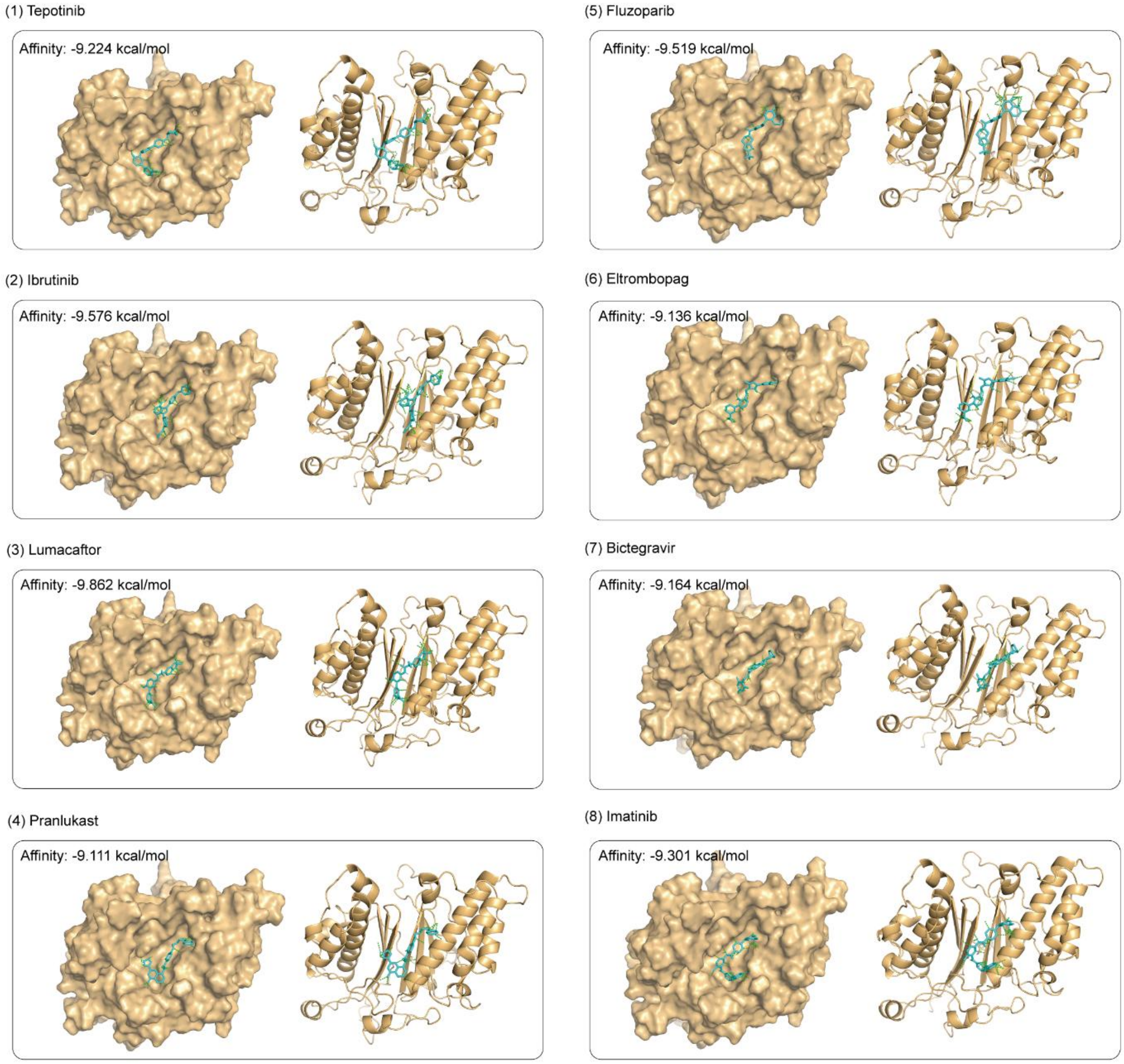

Figure S7. Interaction models of the top 8 FDA-approved drugs with high affinity to PptA. The structures of protein-ligand interaction were visualized by PyMOL software.

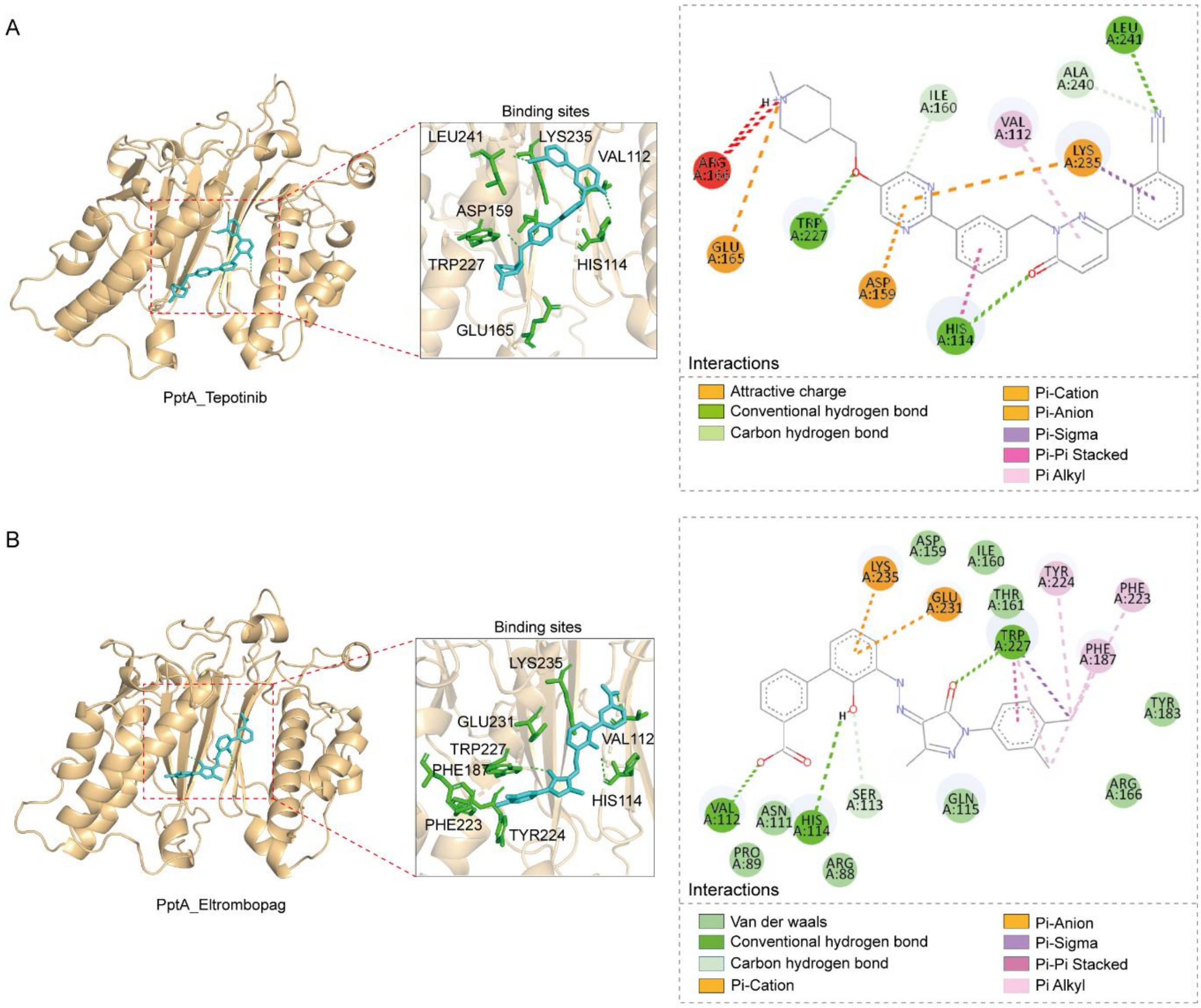

Figure S8. Binding sites analysis of two drug candidates with antifungal activity in PptA. **A-B**) Binding sites and interaction forces of tepotinib (**A**) and eltrombopag (**B**) in PptA.

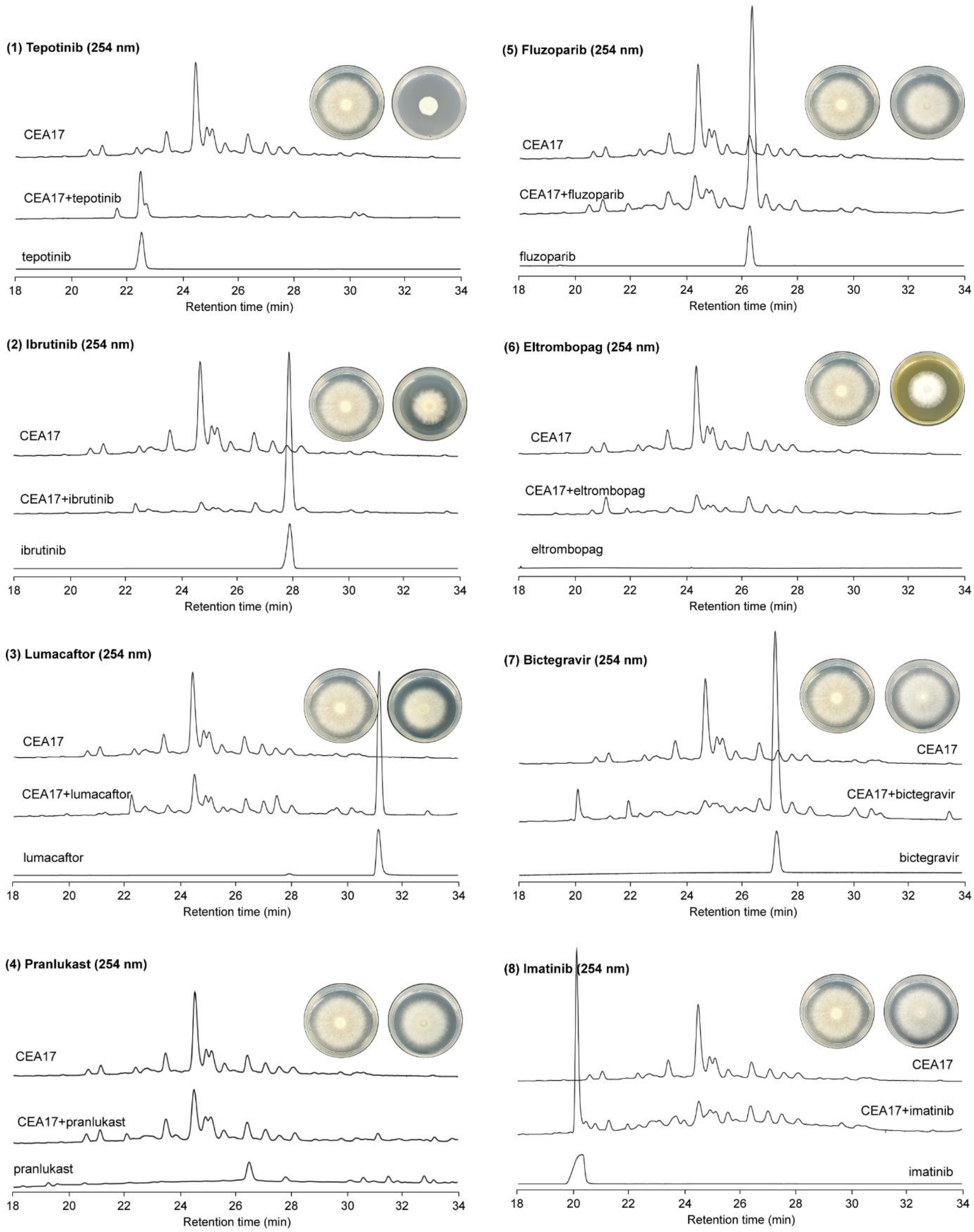

Figure S9. Phenotype and metabolite analysis of *A. fumigatus* CEA17 treated with the top 8 drugs. The phenotype of *A. fumigatus* CEA17 cultured for 3 days and the metabolic profile of *A. fumigatus* CEA17 cultured for 4 days treated with 200 μM drugs.

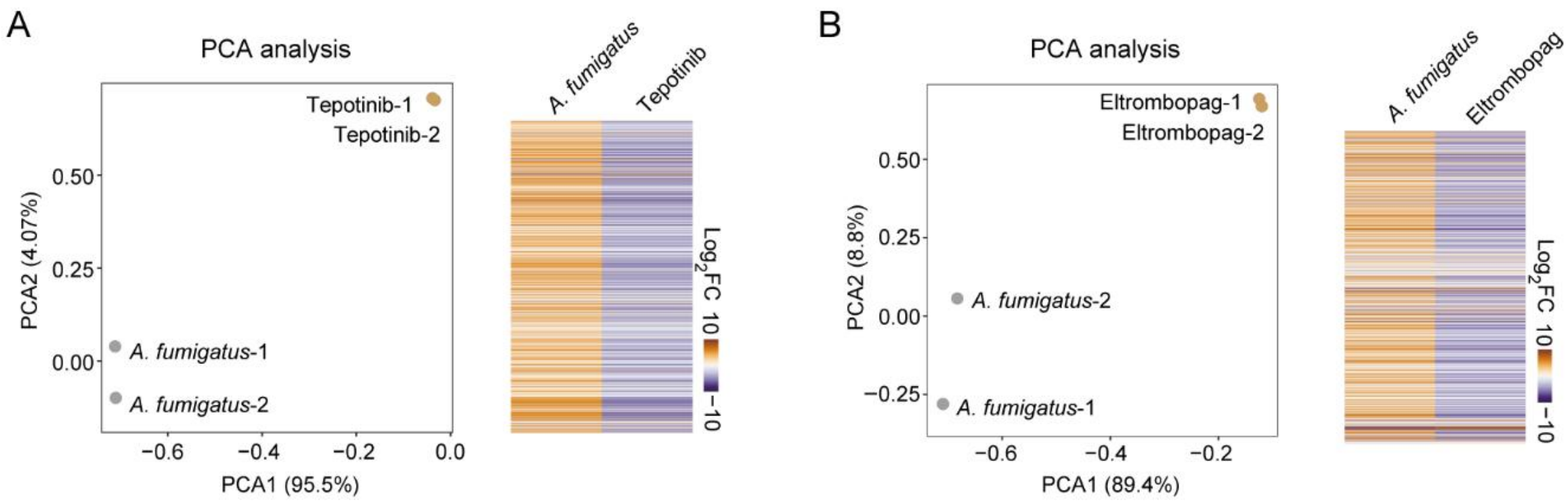

Figure S10. Metabolomic analysis of *A. fumigatus* CEA17 treated with tepotinib and eltrombopag. **A-B**) The metabolic profile of *A. fumigatus* CEA17 cultured for 4 days treated with 200 μM drugs. A Log_2foldchange < −1 indicates repressed metabolic ions in drug-treated groups compared with the control.

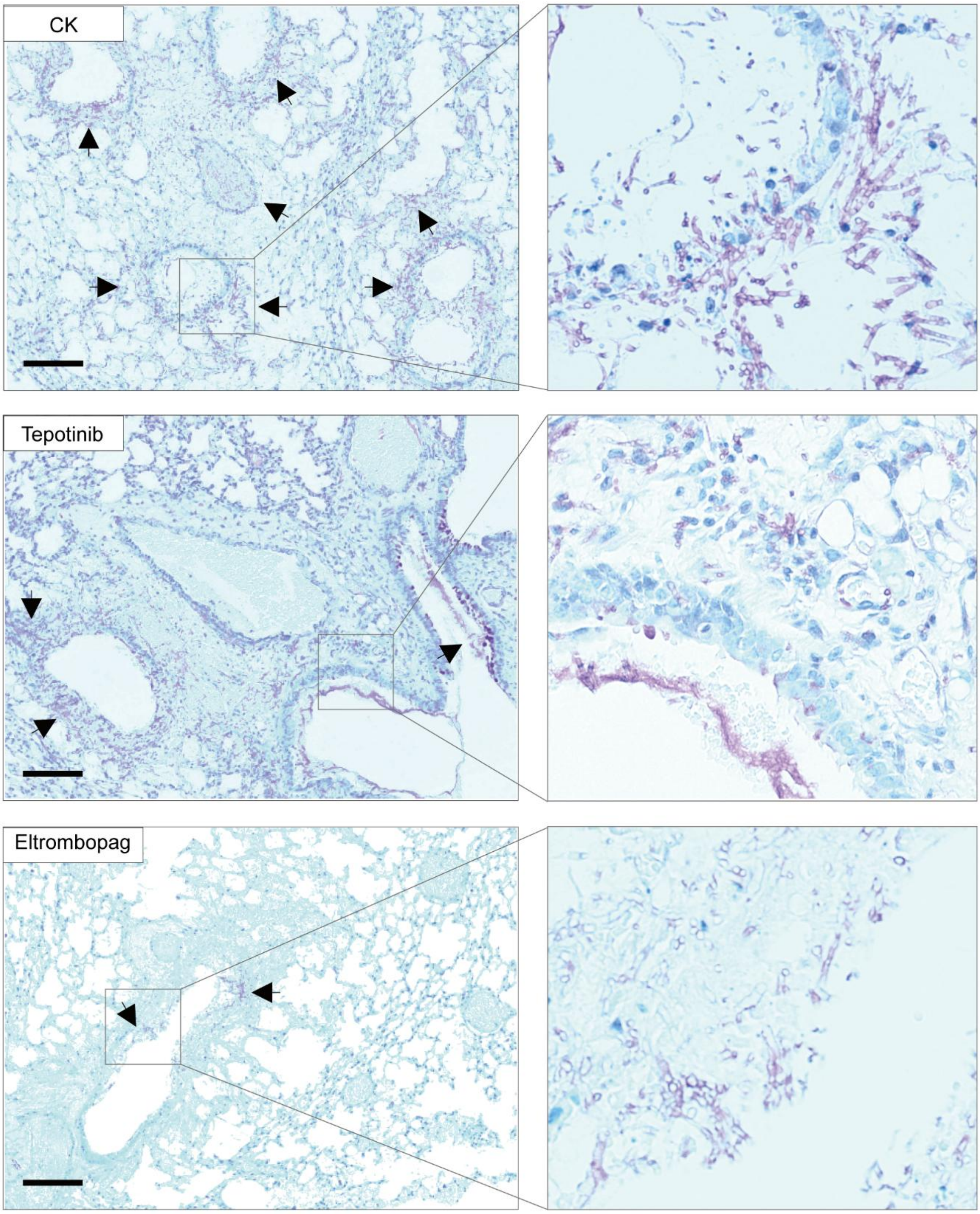

Figure S11. Periodic Acid-Schiff staining of *A. fumigatus* in the mouse lungs after 5 days of treatment with two drug candidates. Scale bars: 25 μm (black arrowheads indicate *A. fumigatus* hyphae). The right panels show the figure with a 5× local magnification.

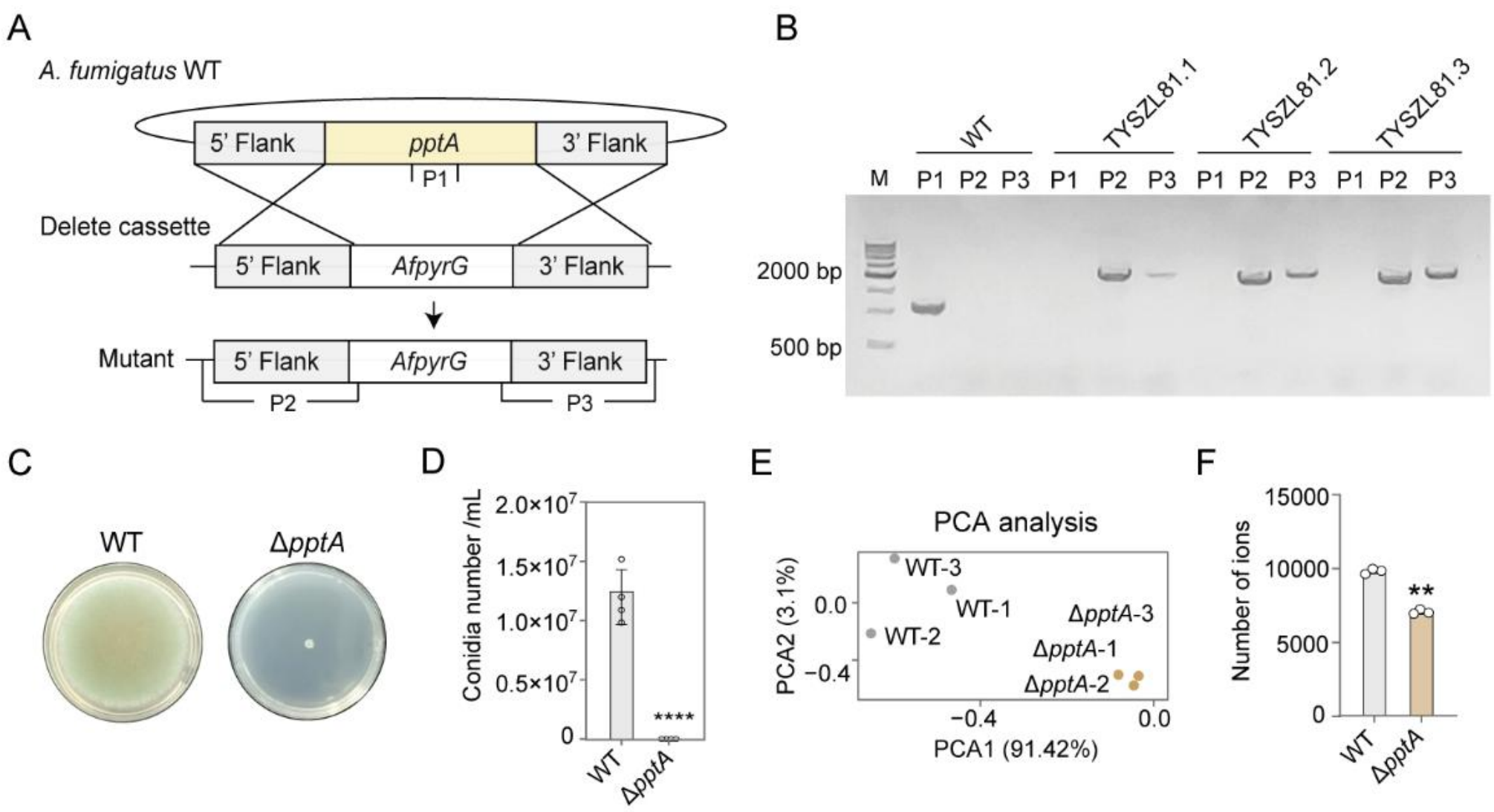

Figure S12. Construction and metabolomics analysis of the Δ*pptA* mutant in *A. fumigatus*. **A**) Schematic diagram of Δ*pptA* knockout in *A. fumigatus*. **B**) PCR validation of the Δ*pptA* mutant. **C**) Colonial phenotype of the Δ*pptA* mutant cultured on GMM medium at 37 °C for 3 days. **D**) Conidial production in *A. fumigatus* and its mutant strains (n = 4). **E**) PCA analysis of metabolic ion profiles between the Δ*pptA* mutant and control strains. **F**) Metabolic ionic products detected in the Δ*pptA* mutant and control strains. A Log_2foldchange < -1 indicates repressed metabolic ions when comparing the metabolic ion abundances between the Δ*pptA* mutant and control strain. All error bars are expressed as mean ± SD. Statistical analysis was performed by using *t*-test (Significant at $^{**}p < 0.01$, $^{****}p < 0.0001$).

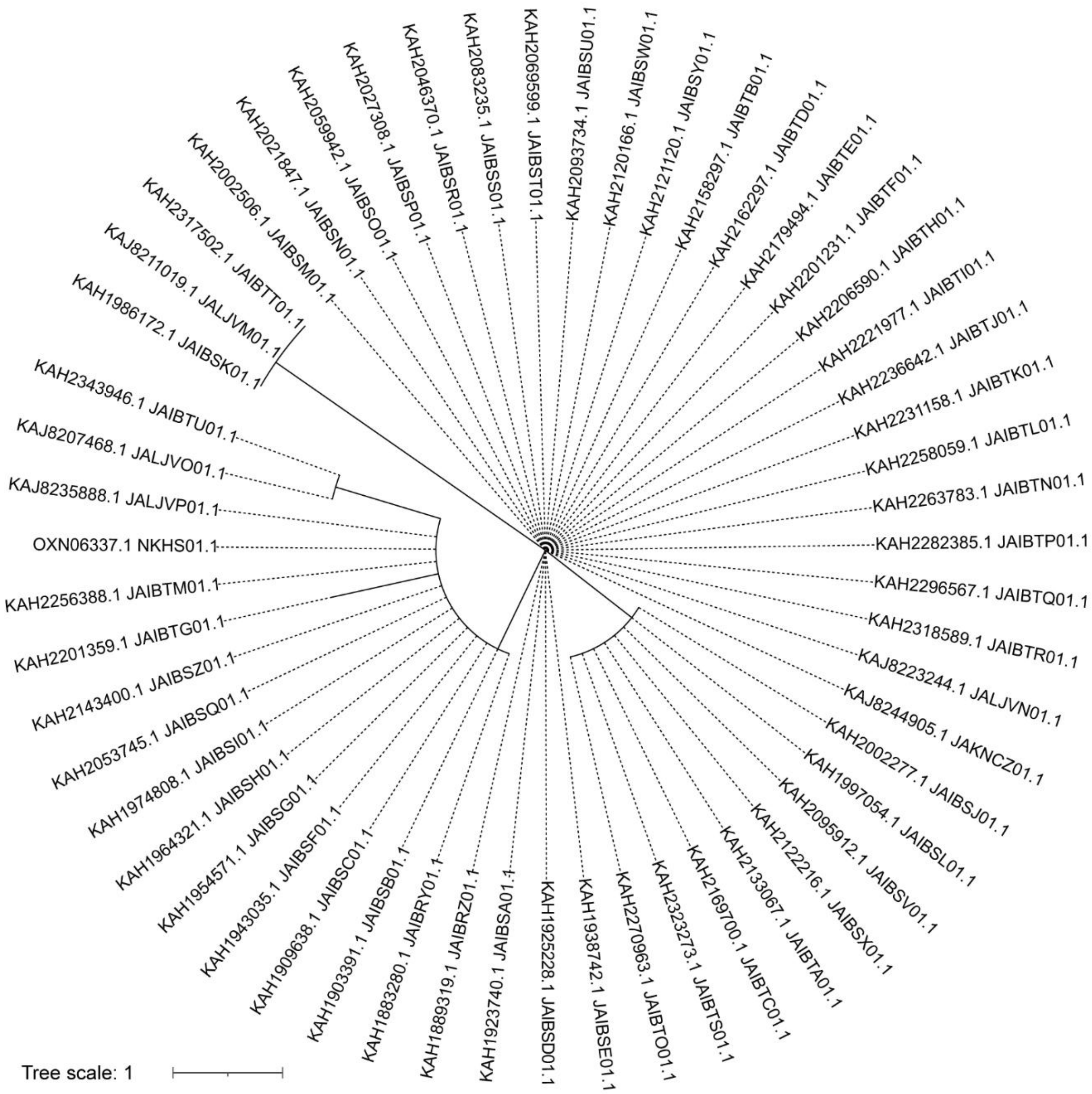

Figure S13. Phylogenetic analysis of the sfp-type PPTases in clinical *A. fumigatus*. The sfp-type PPTase is highly conserved in both azole-resistant and susceptible of clinical *A. fumigatus*.

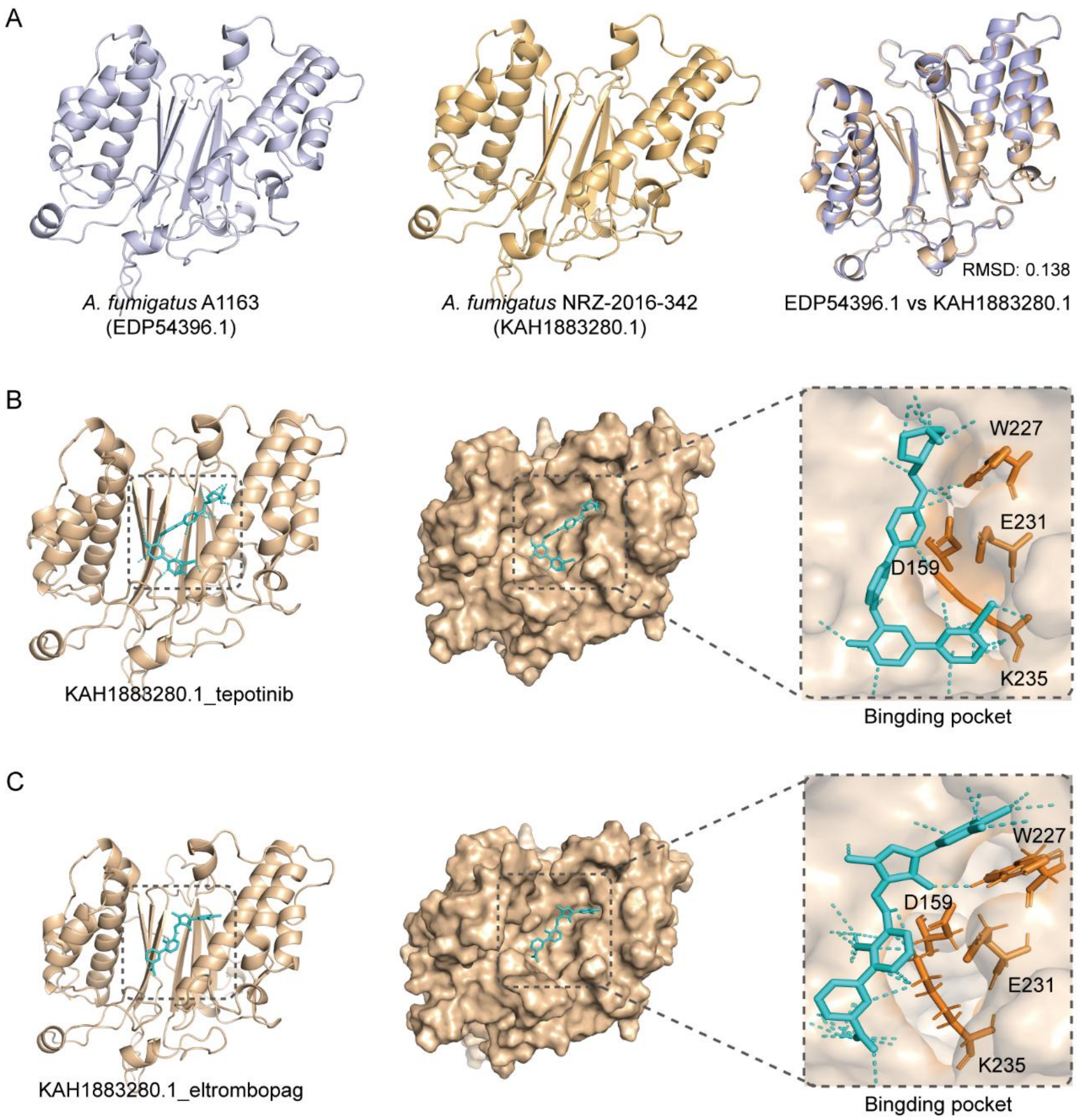

Figure S14. Conservation of PptA structure and drug-binding sites in clinical drug-resistant strain. **A**) Structural conservation of PptA in the clinical drug-resistant *A. fumigatus* NRZ-2016-342. **B-C**) Conservation of tepotinib/eltrombopag binding sites in clinical drug-resistant *A. fumigatus* NRZ-2016-342.

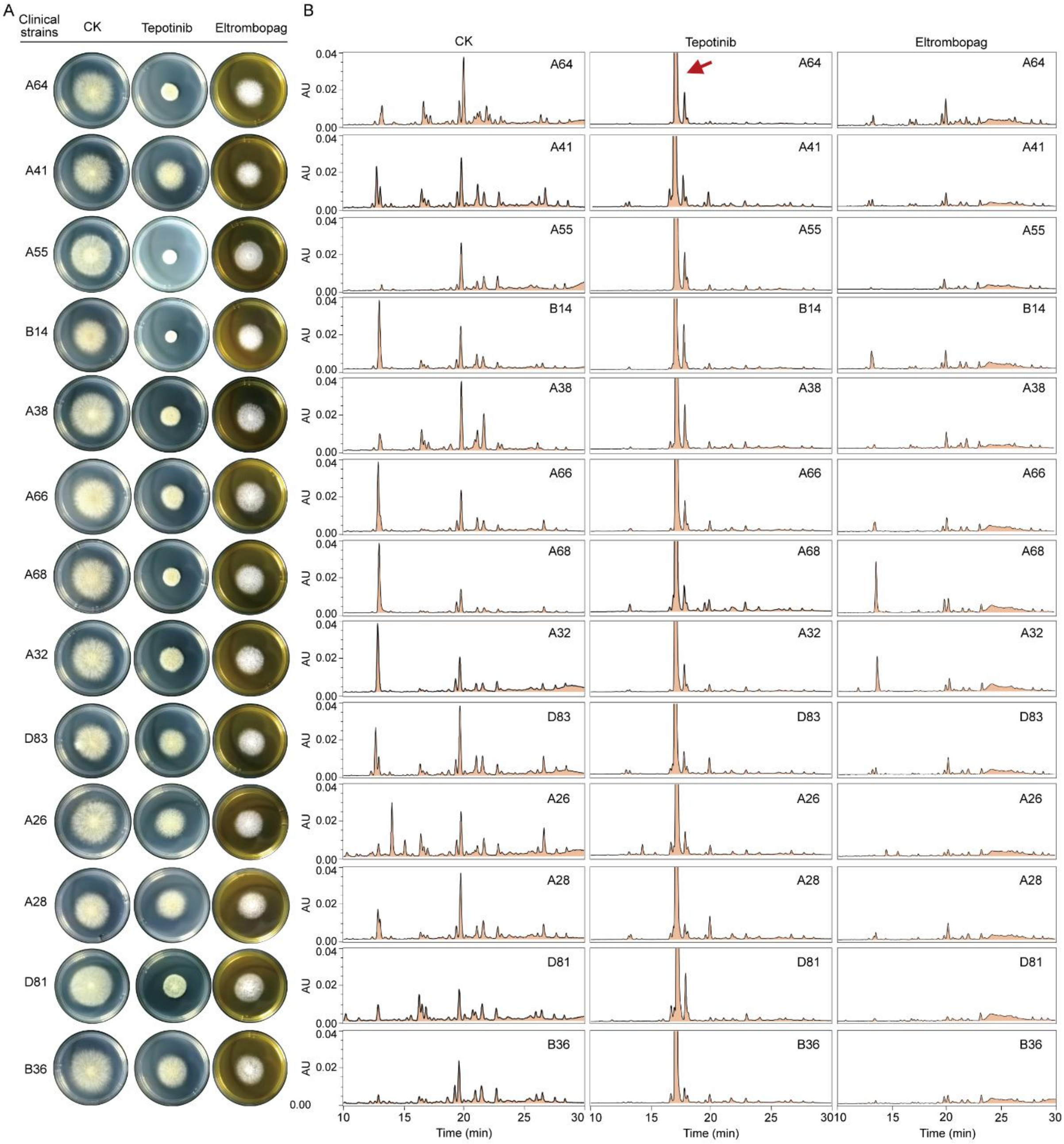

Figure S15. Phenotypic and HPLC analysis of clinical strains treated with 2 drug candidates. **A-B**) The phenotypes and metabolic profiles of clinical *A. fumigatus* cultured for 4 days treated with 200 μM tepotinib or eltrombopag, respectively. The red arrow represents the peak of tepotinib.

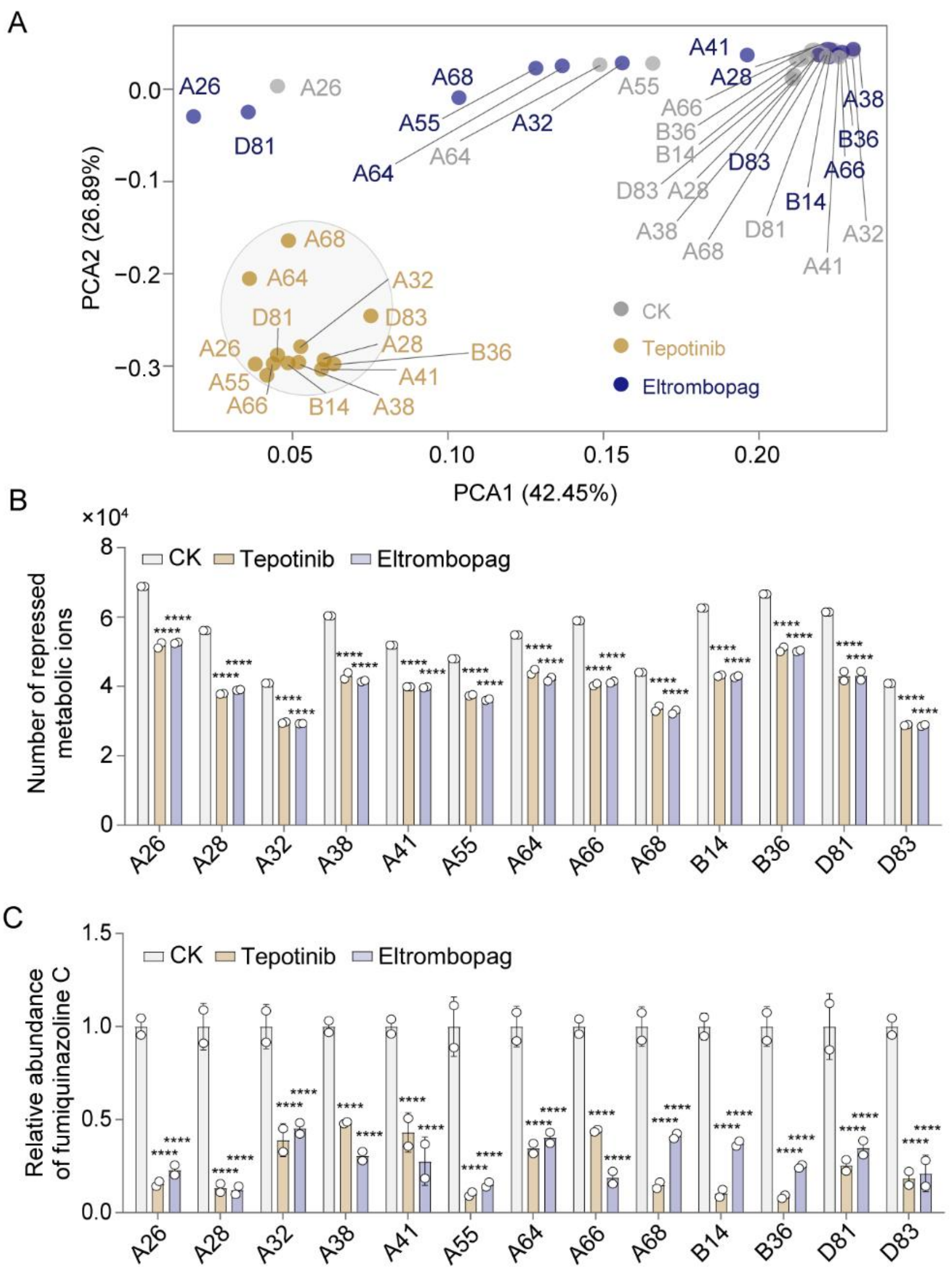

Figure S16. Metabolomic analysis of clinical strains treated with 2 drug candidates. **A**) PCA analysis of metabolomic data from clinical strains treated with tepotinib and eltrombopag. **B**) Total number of suppressed metabolic ions in each clinical strain after treatment with tepotinib and eltrombopag. **C**) Relative abundance of fumiquinazoline C in each clinical strain after treatment with tepotinib and eltrombopag. A Log_2foldchange < −1 indicates repressed metabolic ions in drug-treated groups compared with the control. All error bars are expressed as mean ± SD. Statistical analysis was performed by using Two-way ANOVA (****$p < 0.0001$).

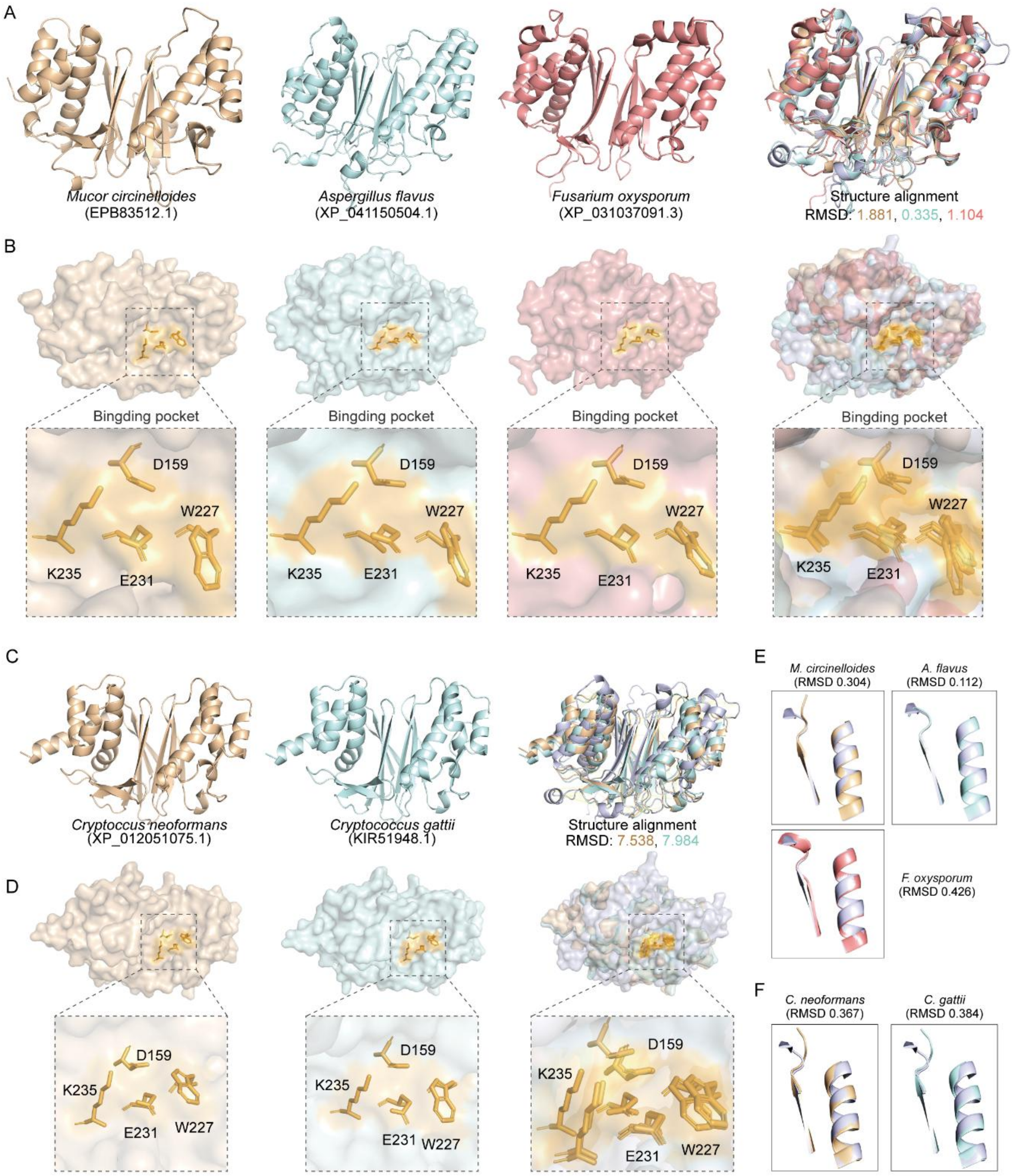

Figure S17. Structural conservation of PPTases across diverse pathogenic fungi. **A-B**) Conservation analysis of structure and active pocket in PPTases across diverse filamentous fungi. **C-D**) Conservation analysis of structure and active pocket in PPTases across diverse *Cryptococcus* species. **E**) Conservation analysis of active-site structure in PPTases across diverse filamentous fungi. **F**) Conservation analysis of active-site structure in PPTases across diverse *Cryptococcus* species.

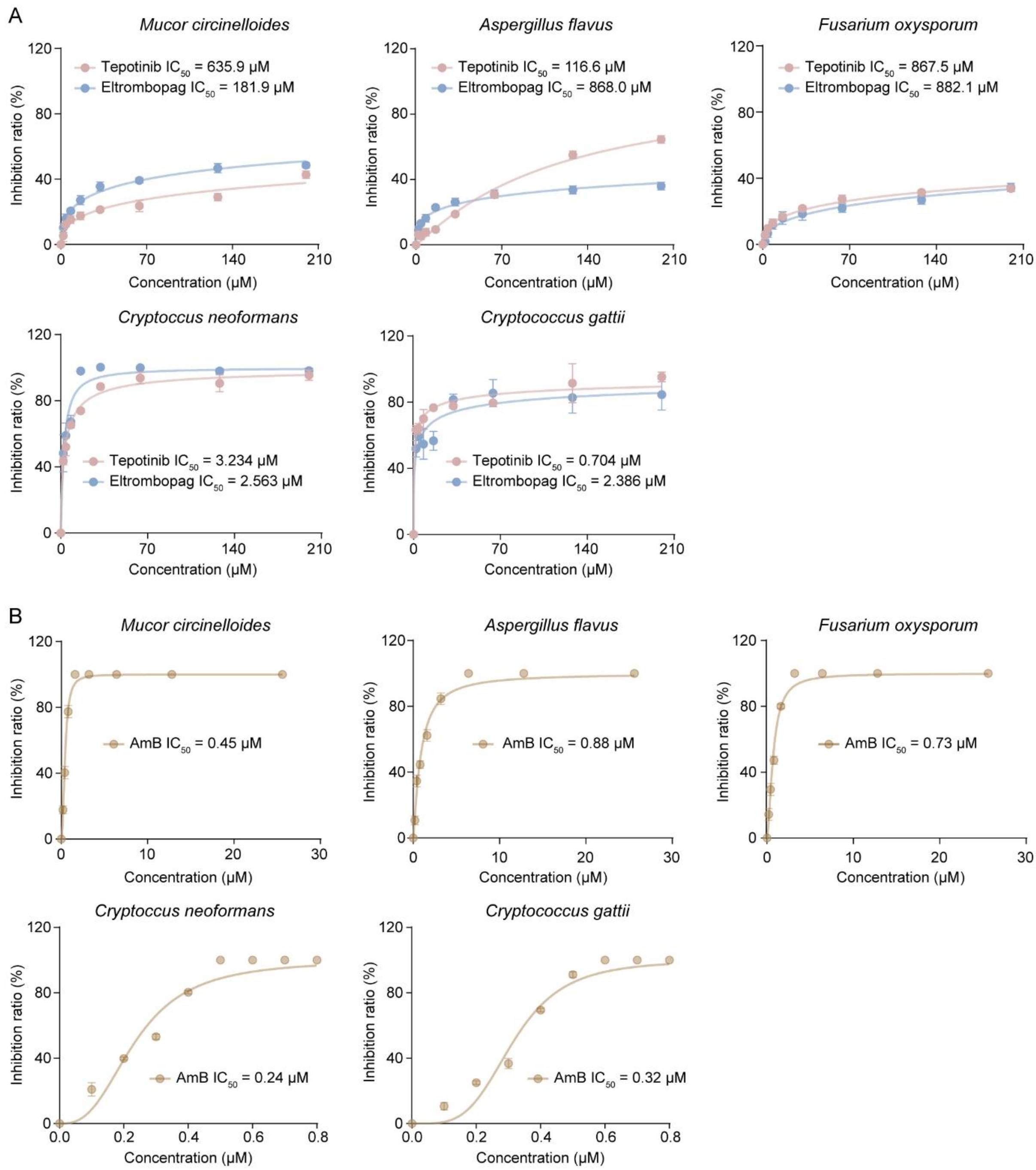

Figure S18. Evaluation of IC_{50} values of two candidate compounds and the positive control AmB against diverse pathogenic fungi. **A**) Evaluation of IC_{50} values of two candidate compounds against diverse pathogenic fungi. **B**) Evaluation of IC_{50} values of positive control AmB against diverse pathogenic fungi. AmB: amphotericin B. IC_{50}: half-maximal inhibitory concentration.

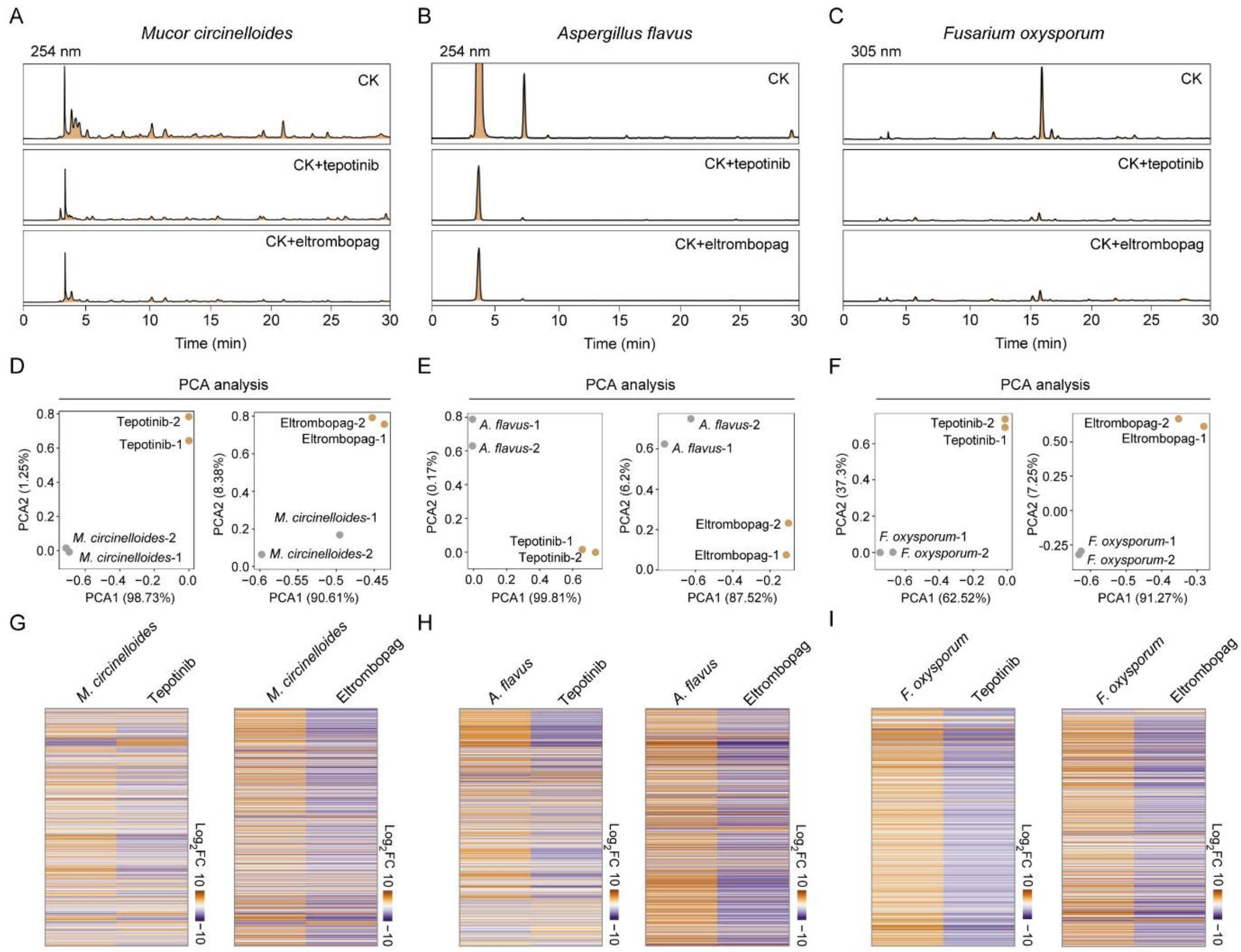

Figure S19. Metabolomic analysis of diverse filamentous fungi treated with tepotinib and eltrombopag. **A-C**) Metabolic profiles of *Mucor circinelloides* (**A**), *Aspergillus flavus* (**B**) and *Fusarium oxysporum* (**C**) treated with tepotinib and eltrombopag. **D-F**) PCA analysis of *M. circinelloides* (**D**), *A. flavus* (**E**) and *F. oxysporum* (**F**) treated with tepotinib and eltrombopag. **G-I**) Heatmap showing metabolomic profiles of *M. circinelloides* (**G**), *A. flavus* (**H**) and *F. oxysporum* (**I**) after tepotinib and eltrombopag treatment. A Log_2foldchange < -1 indicates repressed metabolic ions in drug-treated groups compared with the control.

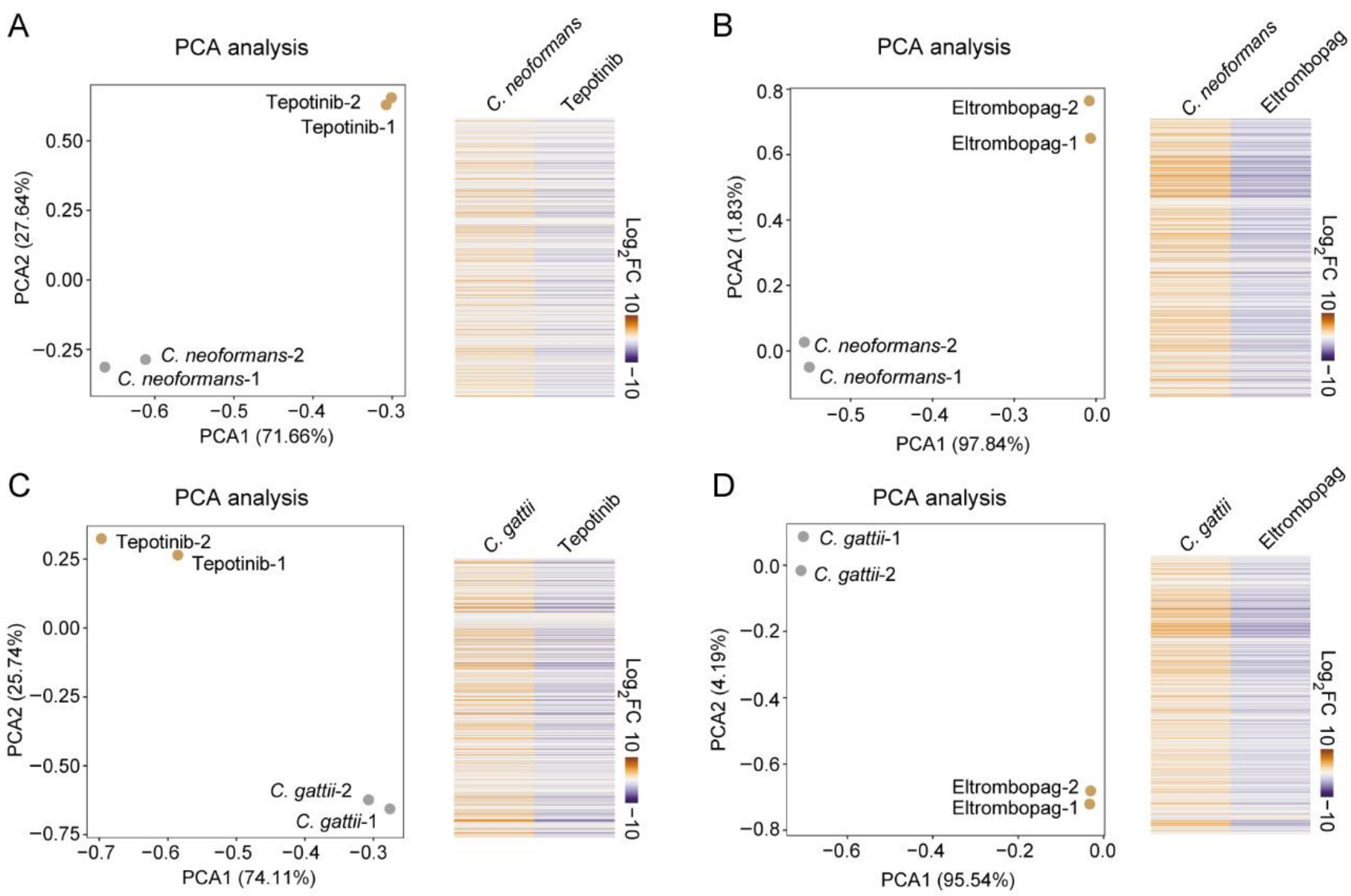

Figure S20. Metabolomic analysis of *Cryptococcus* species treated with tepotinib and eltrombopag. **A-B**) Metabolomic analysis of *C. neoformans* treated with tepotinib (**A**) and eltrombopag (**B**). **C-D**) Metabolomic analysis of *C. gattii* treated with tepotinib (**C**) and eltrombopag (**D**). A Log_2foldchange < −1 indicates repressed metabolic ions in drug-treated groups compared with the control.

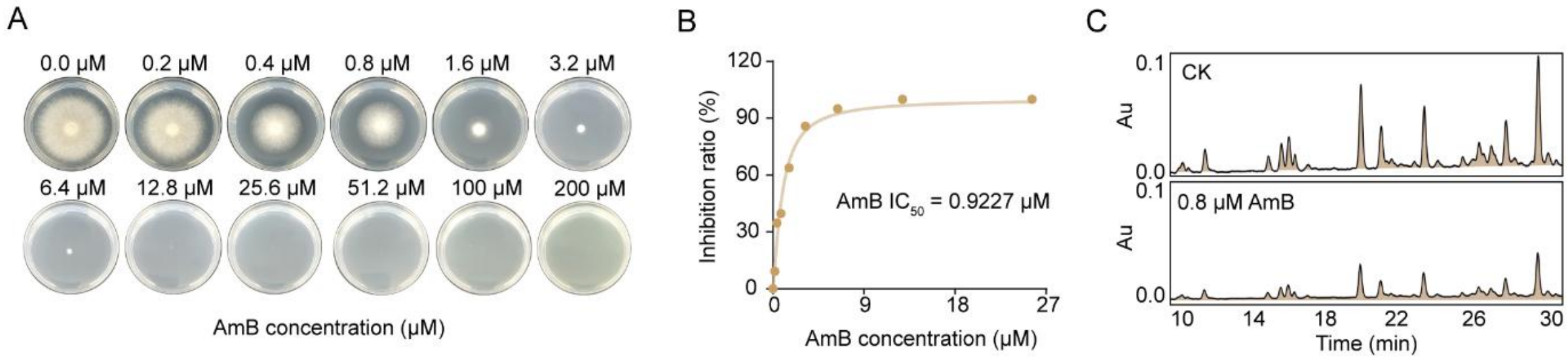

Figure S21. The activity of positive control amphotericin B against *A. fumigatus*. **A-B**) Phenotypes (**A**) and inhibition rates (**B**) of *A. fumigatus* following amphotericin B treatment. **C**) Metabolic profiles of *A. fumigatus* treated with 0.8 μM amphotericin B at 280 nm. AmB: amphotericin B. IC_{50}: half-maximal inhibitory concentration.

Supplementary Tables

Table S1. Fungal species and BGC information.

Table S2. Affinity data for FDA-approved drug with target.

Table S3. Mass spectrometry data of pathogenic fungi.

Table S4. Mass spectrometry data of *pptA* mutant.

Table S5. Mass spectrometry data of clinical *A. fumigatus*.

Table S6. Strains, plasmids and primers.
